# Supplementary material for: PE and PS Lipids Synergistically Enhance Membrane Poration by a Peptide with Anticancer Properties
Source: Biophys J. 2015 Sep 1;109(5):936–47. doi: 10.1016/j.bpj.2015.07.033 (PMC4564682; doi:10.1016/j.bpj.2015.07.033)
Supplement: Document S1. Supporting Materials and Methods and ten figures [file mmc1.pdf]

Supporting Information

PE and PS Lipids Synergistically Enhance Membrane Poration by a Host-Defense Peptide with Anticancer Properties

Natália Bueno Leite[1], Anders Aufderhorst-Roberts[2], Mario Sergio Palma[3], Simon D. Connell[2], João Ruggiero Neto[1,*], Paul A. Beales[4,*]

Author Affiliations:

[1] Department of Physics IBILCE – São Paulo State University -UNESP;

[2] School of Physics and Astronomy and Astbury Centre for Structural Molecular Biology, University of Leeds, Leeds, UK

[3] Center of Studies of Social Insects/Dept. Biology/ IB – São Paulo State University – UNESP.

[4] School of Chemistry and Astbury Centre for Structural Molecular Biology, University of Leeds, Leeds, UK

Brief derivation of the relationship between membrane permeability and fractional permeable area of membrane.

This follows the arguments previously presented by Philip Nelson (Nelson P. 2004. Biological Physics: Energy, Information, Life (Freeman, New York)).

We consider a cylindrical water channel across of biological membrane of length δ, where δ is the bilayer thickness. Initially the concentration of solute (c) outside of the vesicle is c_0 and inside the vesicle is 0. Solute will diffuse through the pore to equilibrate the concentration gradient, Δc, across the membrane. After a brief initial phase of solute influx, a quasi steady state is reached where $dc/dt = 0$. Given the diffusion equation,

$$\frac{dc}{dt} = D_0 \frac{d^2c}{dx^2}$$

where D_0 is the diffusion constant of the solute. This implies that $d^2c/dx^2 = 0$, where x is the spatial coordinate along the length of the pore, parallel to the bilayer normal. This can be solved with the boundary conditions $c(0) = c_0$ and $c(\delta) = 0$ to give $c = c_0(1 - x/\delta)$. By Fick's law, it then follows that the flux $j = -D_0\, \Delta c/\delta$, which can be written as $j = -P_p \Delta c$, where $P_p = D_0/\delta$ is the permeability of a single pore.

If we consider a larger membrane area that might have multiple pores, the diffusive transport across the membrane will only occur through the fraction of the total membrane area (α) that is porous. Therefore the permeability of the membrane, $P_m = \alpha D_0/\delta$, which can be rearranged to give

$$\frac{A_p}{A_v} = \frac{P_m}{D_0}\delta$$

Where A_p is the total area of pores on a vesicle of area A_v.

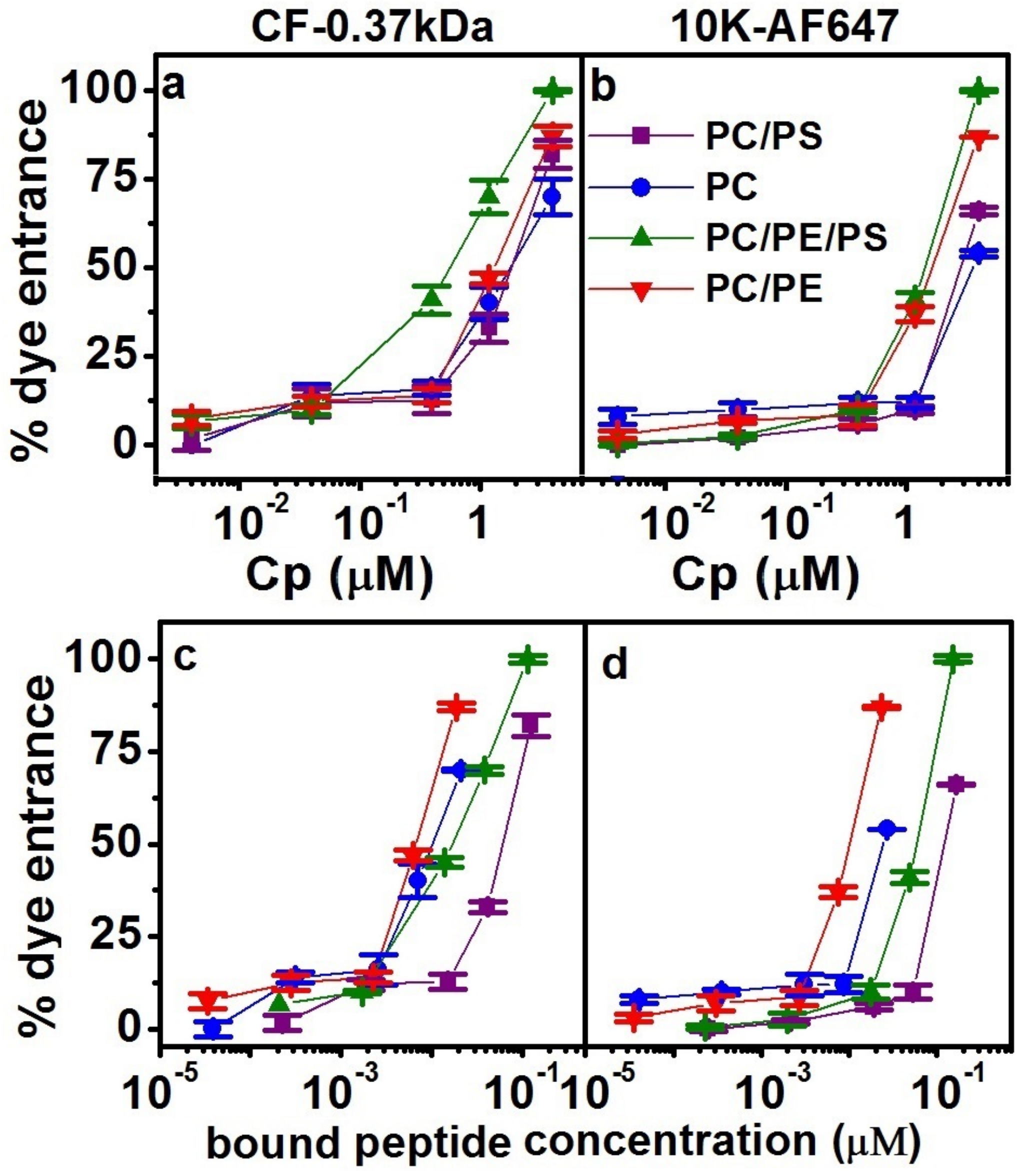

Figure S1. GUV leakage data plotted as average % dye leakage across the GUV population against (a, b) total peptide concentration, and (c, d) peptide concentration bound to the membranes for the different membrane compositions studied. (a, c) leakage of the CF probe; (b, d) leakage of the 10k-AF647 probe. The error bars were obtained through the mean standard deviation of the observed GUVs set.

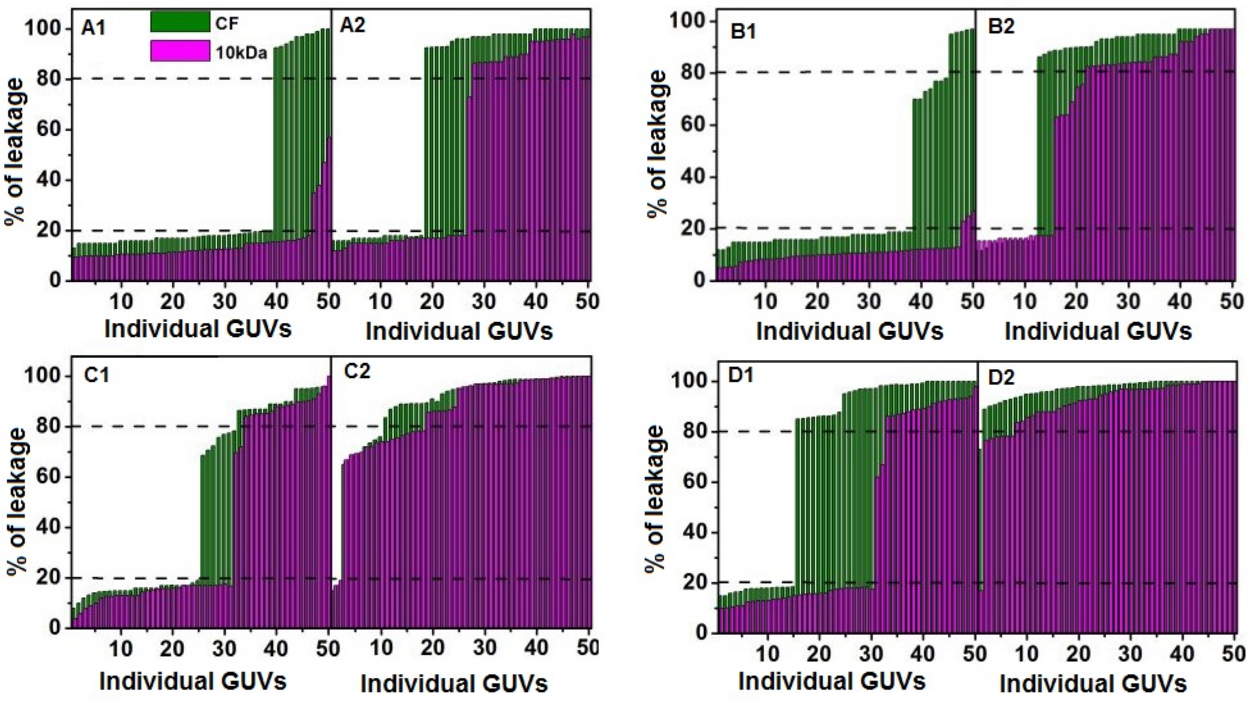

Figure S2 – GUV leakage histograms. Fractional leakage histograms for 50 GUVs per lipid composition after 30 min. incubation with (1) 1.2 µM and (2) 4.0 µM of MP1. Individual GUVs are ordered left to right from lowest to highest leakage %. Lipid compositions are (A) PC, (B) PC/PS, (C) PC/PE and (D) PC/PE/PS. Leakage is analysed for the CF (green) and 10k-AF647 (magenta) probes. Horizontal lines at 20% and 80% leakage represent the thresholds used to categorized leaked vesicles and fully leaked vesicles, respectively. Note that partially leaked vesicles are comparatively rare for these peptide – membrane systems.

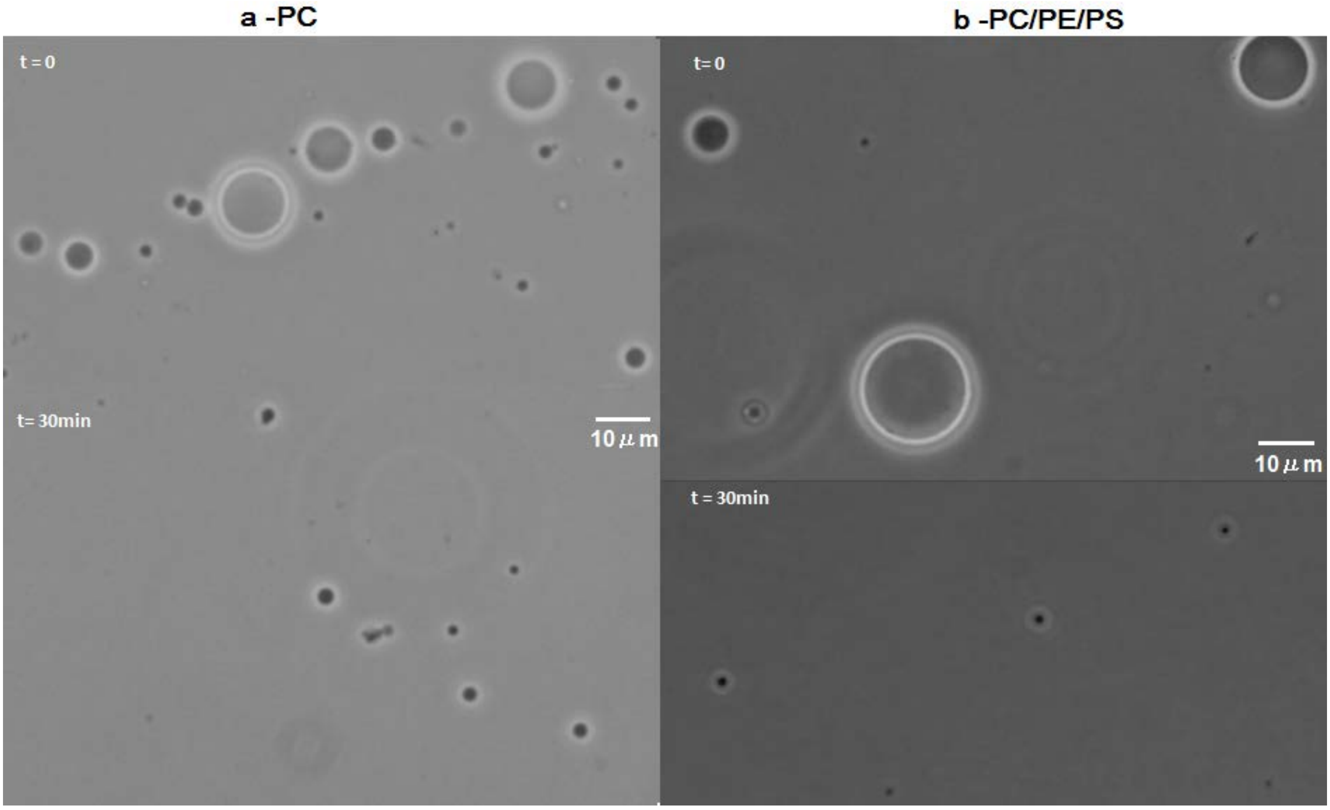

Figure S3 - Phase Contrast of GUVs. Peptide solution was added to GUVs solution to reach a final concentration of 10 µM. Before the peptide addition, at t=0, GUVs were observed in the sample. After the peptide addition, the GUVs are observed to have been completed disrupted (lysed) after 30 min. a- GUVs composed of PC are shown in t=0, and 30 min after peptide addition in the same region of the sample; almost no more GUVs are observed. b- GUVs composed of PC/PE/PS are shown in t=0, and 30 min after peptide addition at the same region; almost no more GUVs are observed.

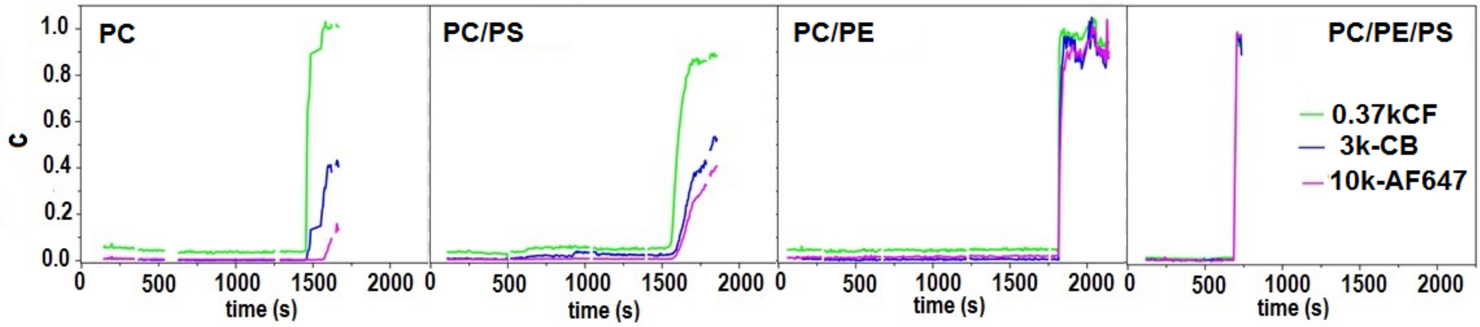

Figure S4. Example profiles for the typical kinetics of single vesicle permeation due to the addition of MP1 at 4.0µM final concentration in four lipid compositions: by PC, PC/PS, PC/PE and PC/PE/PS.

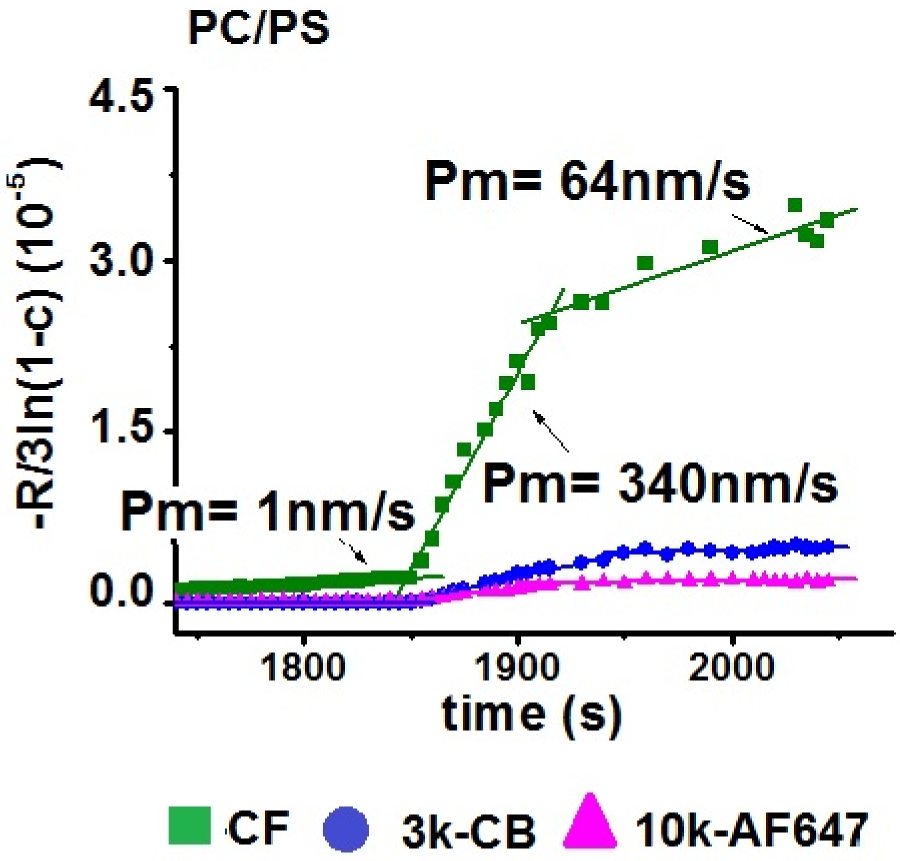

Figure S5. A permeability plot for a PC/PS GUV that clearly exhibits significant changes in membrane permeability during the initial leakage events. The permeability to the CF probe changes dynamically from 1 nm/s to 340 nm/s to 64 nm/s with abrupt transitions between these stages.

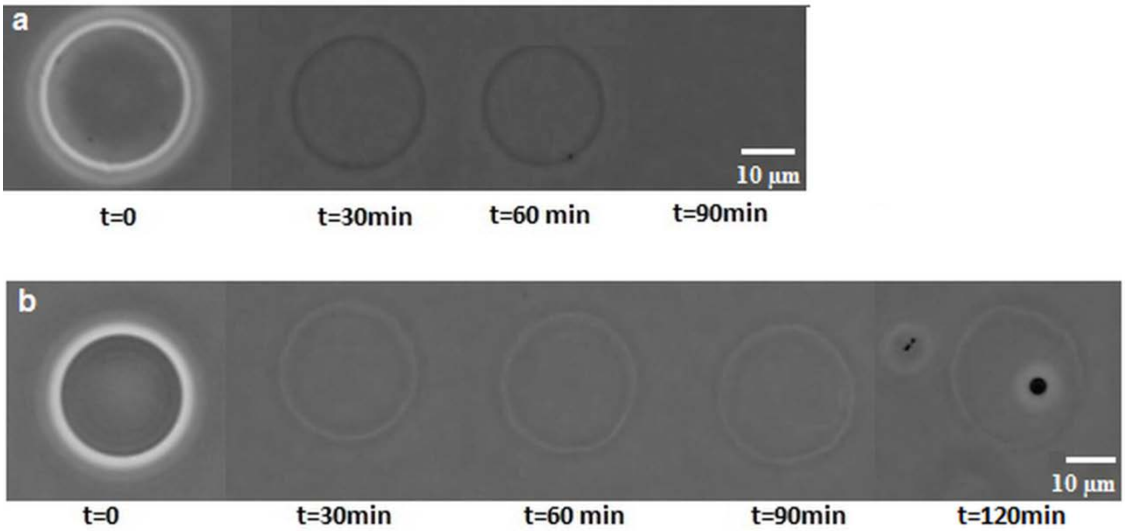

Figure S6 - Phase contrast images of GUVs composed of (a) PC/PE/PS and (b) PC/PS are obtained for a period of 2 h. after addition of 4.0 μM MP1. a- At t=30min most of the phase contrast of the GUV is lost, due to leakage of the encapsulated sucrose solution; the vesicle structure integrity is kept intact for ~1 h. after the contrast is lost. The vesicle size decreased with increasing time of peptide interaction according to: at t ≈ 0 the vesicle diameter (d) was 19 ± 1 µm; at t ≈ 30 min., d= 18 ± 1 µm; at t ≈ 60 min., d = 17 ± 2 µm; and at t ≈ 85 min. the vesicle had lost its integrity. b- At t = 30 min. most of the phase contrast is lost from the GUV and the vesicle structure integrity remains intact over 2 h. after the contrast decreasing. The vesicle size does not changed with increasing time of peptide interaction; the vesicle diameter remained at 25 µm during the observation time.

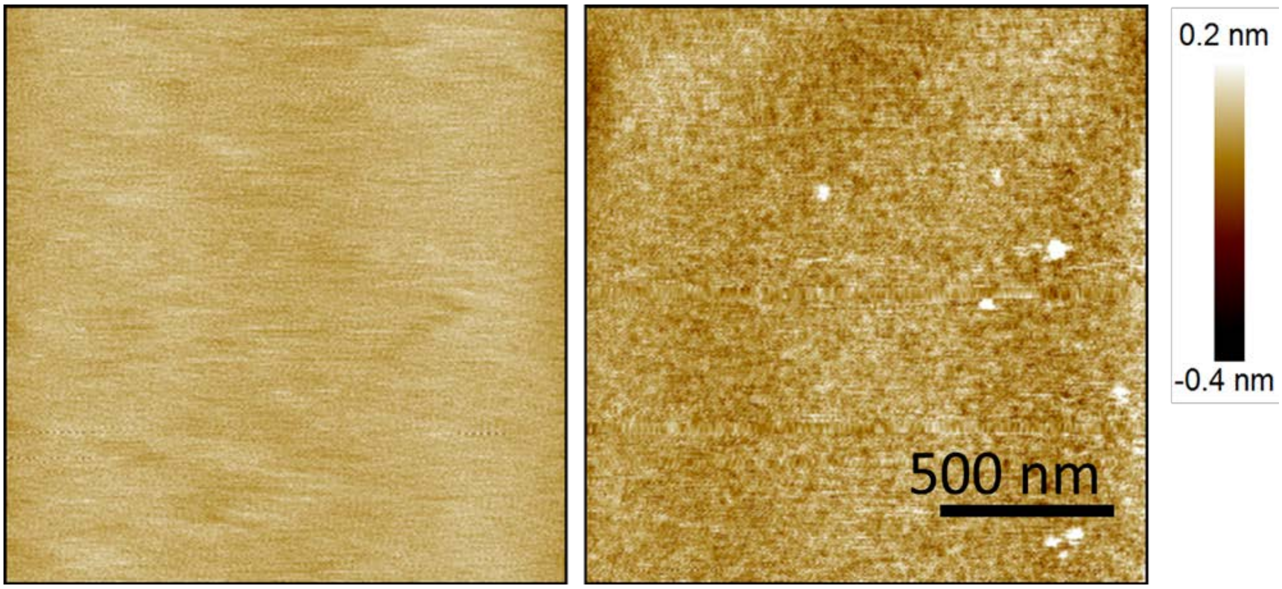

Figure S7. AFM images of a DOPC membrane 80 min. after addition of 10 μM MP1. No clear defects can be observed, but a textured bilayer surface can be seen if the z-contrast is enhanced by a factor of 10. This could indicate a layer of absorbed peptide.

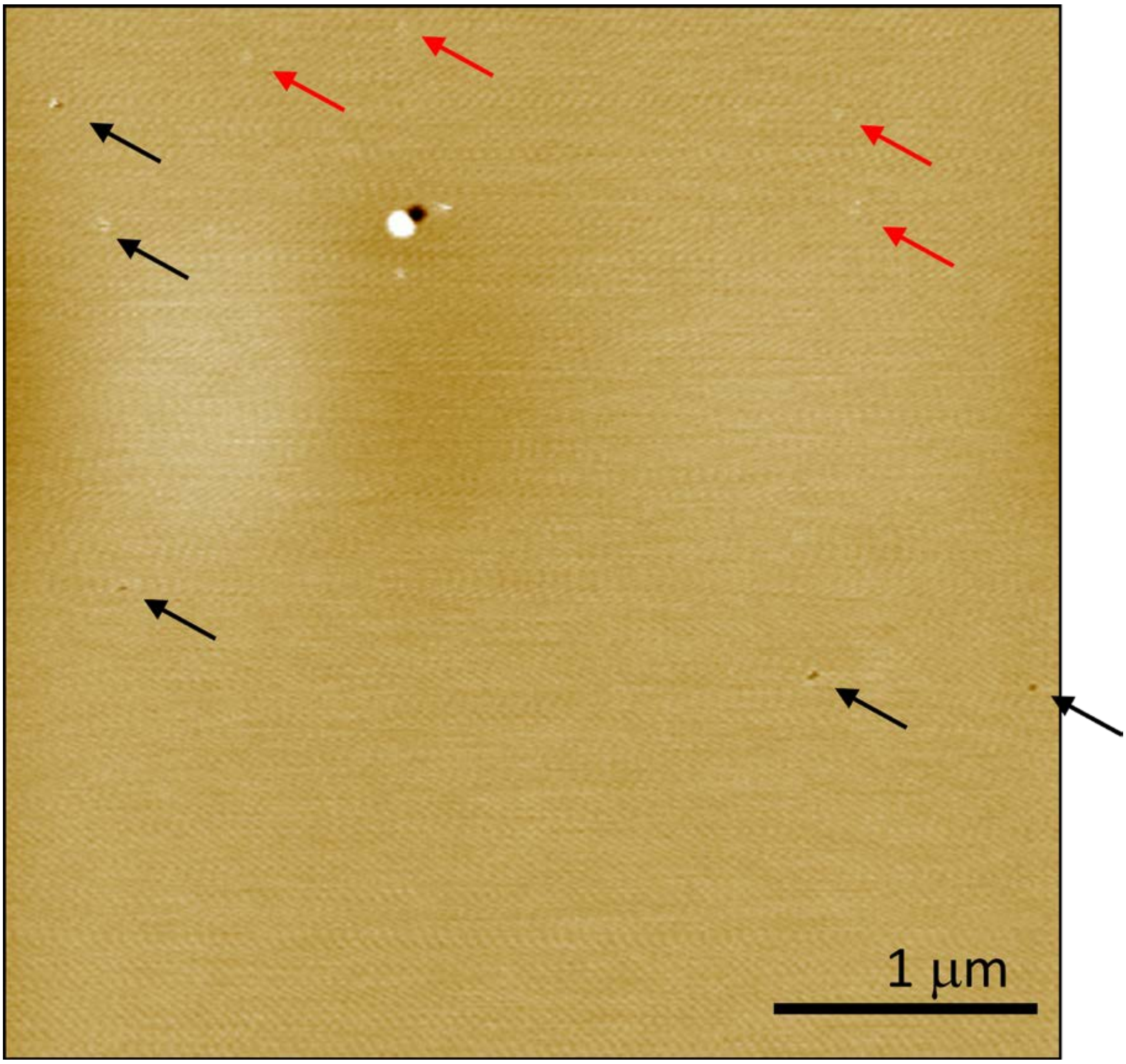

Figure S8. AFM image showing 10-20 nm pin-holes (black arrows) in PC/PE/PS membranes in the first scan, 5 min. after addition of 10 µM MP1. In addition feint “blisters” (some depicted by red arrows) can be seen, which will later develop into pores. A larger pore can be seen top centre-left with a vesicle forming at the edge; vesicle micellisation at the pore rim was observed in time lapse sequences to be the quantized growth mechanism of larger pore defects.

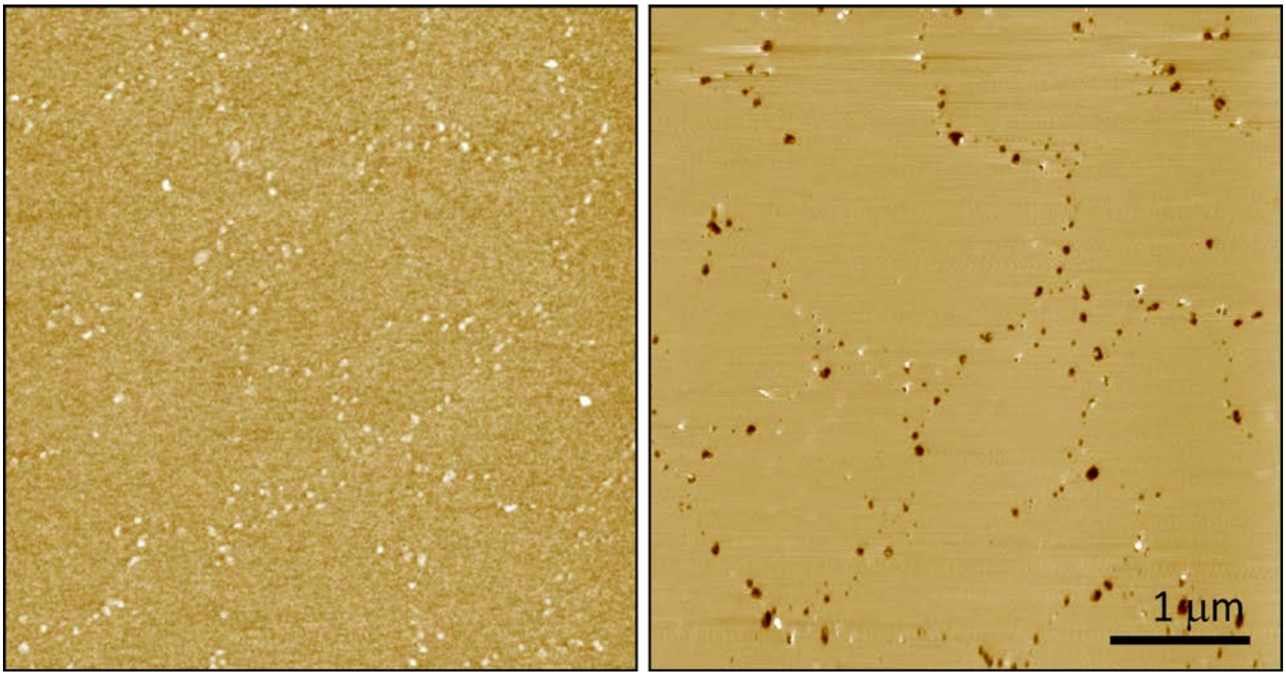

Figure S9. AFM images of PC/PS membranes. (Left) 5-10 min. after addition of 10 μM MP1 "blisters" can be observed in the membrane that will eventually form pores. (Right) Pores observed in the membrane at 2 h after peptide addition. Interestingly, note that the blisters and pores appear to form along a network of lines on the membrane, implying that there may be some cooperativity between the occurrences of neighboring membrane defects.

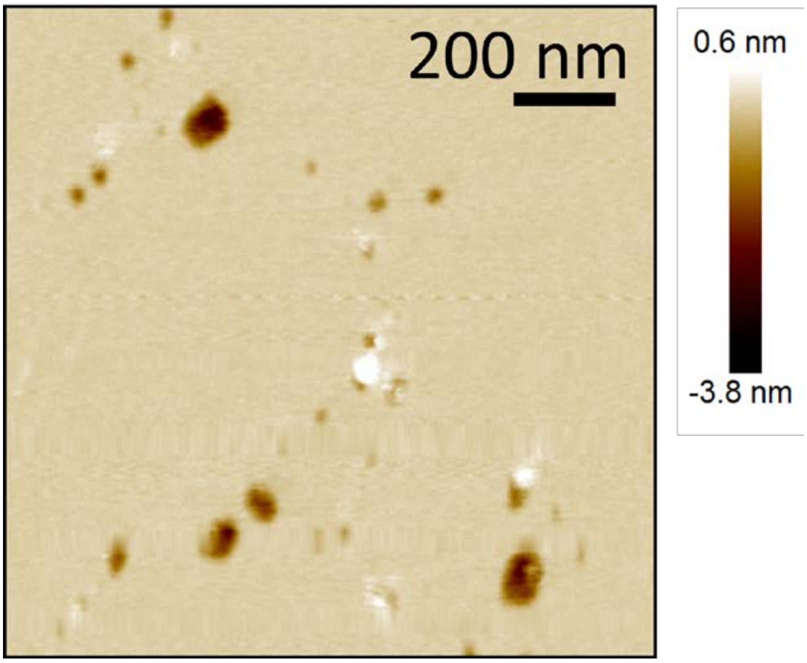

Figure S10. AFM image showing the structural details of pores in PC/PS membranes. Half-bilayer defects (mid-brown) can be observed, along with pores containing full-bilayer pores in their centre (black).
