# Supplementary material for: PE and PS Lipids Synergistically Enhance Membrane Poration by a Peptide with Anticancer Properties
Source: Biophys J. 2015 Sep 1;109(5):936–47. doi: 10.1016/j.bpj.2015.07.033 (PMC4564682; doi:10.1016/j.bpj.2015.07.033)
Supplement: Document S2. Article plus Supporting Material [file mmc2.pdf]

Article

PE and PS Lipids Synergistically Enhance Membrane Poration by a Peptide with Anticancer Properties

Natália Bueno Leite,[1] Anders Aufderhorst-Roberts,[2] Mario Sergio Palma,[3] Simon D. Connell,[2] João Ruggiero Neto,[1,*] and Paul A. Beales[4,*]

[1]Department of Physics, Instituto de Biociências Letras e Ciências Exatas, São Paulo State University, Universidade Estadual Paulista "Júlio de Mesquita Filho", São Paulo, Brazil; [2]School of Physics and Astronomy and Astbury Centre for Structural Molecular Biology, University of Leeds, Leeds, UK; [3]Center of Studies of Social Insects and Department of Biology and Instituto de Biociências, São Paulo State University, Universidade Estadual Paulista "Júlio de Mesquita Filho", São Paulo, Brazil; and [4]School of Chemistry and Astbury Centre for Structural Molecular Biology, University of Leeds, Leeds, UK

ABSTRACT Polybia-MP1 (MP1) is a bioactive host-defense peptide with known anticancer properties. Its activity is attributed to excess serine (phosphatidylserine (PS)) on the outer leaflet of cancer cells. Recently, higher quantities of phosphatidylethanolamine (PE) were also found at these cells' surface. We investigate the interaction of MP1 with model membranes in the presence and absence of POPS (PS) and DOPE (PE) to understand the role of lipid composition in MP1's anticancer characteristics. Indeed we find that PS lipids significantly enhance the bound concentration of peptide on the membrane by a factor of 7–8. However, through a combination of membrane permeability assays and imaging techniques we find that PE significantly increases the susceptibility of the membrane to disruption by these peptides and causes an order-of-magnitude increase in membrane permeability by facilitating the formation of larger transmembrane pores. Significantly, atomic-force microscopy imaging reveals differences in the pore formation mechanism with and without the presence of PE. Therefore, PS and PE lipids synergistically combine to enhance membrane poration by MP1, implying that the combined enrichment of both these lipids in the outer leaflet of cancer cells is highly significant for MP1's anticancer action. These mechanistic insights could aid development of novel chemotherapeutics that target pathological changes in the lipid composition of cancerous cells.

INTRODUCTION

The antimicrobial peptide Polybia-MP1 (IDWKKLLDAAK QIL-NH2), or simply MP1, has unexpectedly been shown to exhibit selective inhibition against several types of cancerous cells and therefore could prove advantageous in the development of novel chemotherapies. Extracted from the Brazilian wasp *Polybia paulista*, MP1 has a broad spectrum of bactericidal activities against Gram-negative and Gram-positive bacteria without being hemolytic and cytotoxic (1). Surprisingly, MP1 also selectively inhibits proliferating bladder and prostate cancer cells (2), and multidrug-resistant leukemic cells (3). Recently, it has been observed that this peptide is cytotoxic against leukemic T lymphocytes and very selective in recognizing these cells compared to healthy lymphocytes (4).

Cancer cell membranes are now known to lose the asymmetric transmembrane distribution of phospholipids that is observed in healthy cells (5,6). In healthy mammalian cells, the anionic aminophospholipid PS (phosphatidylserine) is predominant in the inner membrane leaflet and zwitterionic phospholipids are predominant in outer membrane leaflet. In such cells, the phospholipid asymmetry is maintained by a family of aminophospholipid translocases that catalyze the transport of PS from the outer to the inner membrane leaflets (7). However, in apoptotic and cancer cells, PS is found to also be located in the outer monolayer of the plasma membrane in significant proportions (5,6).

The molecular-scale mechanistic basis for MP1's anticancer properties is yet to be established. Changes in the distribution and/or composition of lipids (e.g., PS) within the plasma membrane of malignant cells could be the origin of MP1's cancer selectivity. This is a reasonable hypothesis, based upon the well-established selectivity of antimicrobial peptides for bacterial membranes over eukaryotic membranes due to their higher anionic lipid content (8–11). Recently, the effect of PS on the pore-forming activity of MP1 was investigated by multiple techniques, namely, conductance measurements in planar bilayer lipid membranes, binding assays, and lytic activity on large unilamellar vesicles (4). Although an increase in affinity and lytic activity of MP1 for lipid vesicles containing PS was observed, MP1's pore-formation activity in BLM showed no difference between PC (phosphatidylcholine) and mixed PC/PS bilayers. Significantly, it was recently reported that PE (phosphatidylethanolamine) lipids, naturally found on the inner plasma membrane of normal cells, are also externalized to the outer monolayer of the plasma membrane of

Submitted January 30, 2015, and accepted for publication July 22, 2015.

*Correspondence: p.a.beales@leeds.ac.uk or jruggiero@sjrp.unesp.br

This is an open access article under the CC BY license (http://creativecommons.org/licenses/by/4.0/).

Editor: Paulo Almeida.

© 2015 by the The Authors

0006-3495/15/09/0936/12

http://dx.doi.org/10.1016/j.bpj.2015.07.033

apoptotic and tumor endothelial cells due to both PS and PE lipids being coregulated by the same transporters (7). These authors observed that the exposure to the outer monolayer of one of these phospholipids leads to the exposure of the other. Therefore, it is important for future work to establish the role of increased concentrations of both PE and PS lipids in the interaction of MP1 with membranes.

In this work, we address this challenge by establishing the roles of PE and PS lipids in the effects of MP1 on the structure and permeability of model membranes. Primarily, we study the permeability of giant unilamellar vesicles (GUVs) at the single vesicle level. Fluorescence confocal microscopy was used to determine the size-dependent macromolecular permeability of lipid membranes in GUV model systems by analyzing the influx of three fluorescent dyes with molecular masses of 0.37, 3.0, and 10.0 kDa into these vesicles (Fig. 1). We deconvolve the effects of PS and PE lipids by exploring their effects within DOPC (PC) membranes both separately and in combination: DOPC/POPS 80:20 (PC/PS), DOPC/DOPE 90:10 (PC/PE), and DOPC/DOPE/POPS 70:10:20 (PC/PE/PS). These experiments are corroborated by circular dichroism (CD) spectroscopy to quantify peptide binding to the membrane, fluorescence spectroscopy experiments to establish the leakage mechanism in an ensemble system of nanoscale large unilamellar vesicles (LUVs), and atomic-force microscopy (AFM) imaging of supported lipid bilayers to reveal the nanoscale perturbations of membrane structure induced by the peptide. By combining these approaches, we show that, while PS lipids significantly enhance MP1's binding onto the membrane, PE lipids impart the most significant contribution to the rate and extent of membrane permeabilization by MP1, facilitating the opening of larger membrane defects than in bilayers lacking in PE.

MATERIALS AND METHODS

Materials

DOPC (1,2-dioleoyl-*sn*-glycero-3-phosphocholine), DOPE (1,2-dioleoyl-*sn*-glycero-3-phosphoethanolamine), POPS (1-palmitoyl-2-oleoyl-*sn*-glycero-3-phosphoserine), and Rh-DOPE (1,2-dioleoyl-*sn*-glycero-3-phosphoethanolamine-*n*-(lissamine rhodamine B sulfonyl) (ammonium salt) were purchased from Avanti Polar Lipids (Alabaster, AL). A quantity of 10 kDa Alexa Fluor 647-labeled dextran (10k-AF647), 3 kDa Cascade Blue-labeled dextran (3k-CB), ANTS (8-aminonaphtalene-1, 3, 6-trisulfonic acid sodium salt), and DPX (p-xylenebis (pyridinum) bromide) were purchased from Invitrogen Molecular Probes (Waltham, MA). CF (carboxyfluorescein) (370 MW) and all other reagents were purchased from Sigma-Aldrich (St. Louis, MO). Buffer: 10 mM HEPES and 150 mM NaCl, pH 7.4.

Peptide synthesis and purification

MP1 (Polybia-MP1) was synthesized, as described in de Souza et al. (1,12) by stepwise manual solid-phase synthesis using Fmoc (N-9-fluorophenylmethoxycarbonyl) chemistry. The crude product was purified by reverse-phase HPLC and the homogeneity and sequence was assessed by analytical HPLC and electrospray ionization (ESI) mass spectrometry.

Mass spectrometry analysis

The homogeneity of peptide preparations were ascertained by mass spectrometry. Samples were analyzed on a triple quadrupole mass spectrometer, model QUATTRO II, equipped with a standard ESI probe (Waters/Micromass,

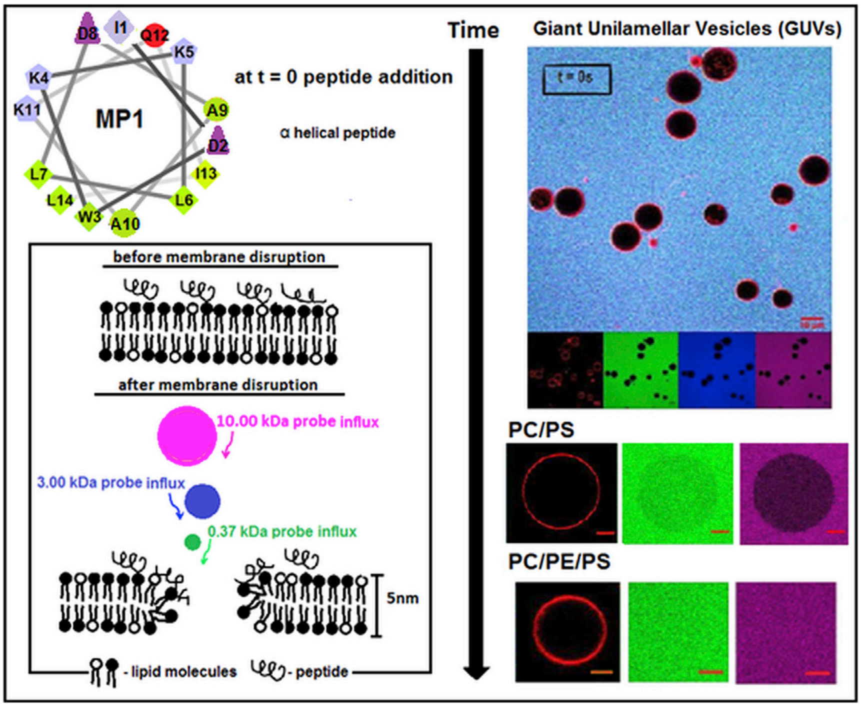

FIGURE 1 Schematic representation of membrane disruption by peptides and the experimental system. The helical peptide Polybia-MP1 is shown according to the helical wheel projections. Amino acids: (*blue*) polar with positive net charge; (*purple*) polar with negative net charge; (*red*) polar noncharged; and (*green*) nonpolar. Confocal microscopy was performed to investigate the influx of three dyes with distinct sizes in GUVs in the presence and absence of PE lipids: 0.37 kDa CF (*green*), 3k-CB (*blue*), 10k-AF647 (*magenta*), and the scale bars correspond to 10 μm. Lipid membranes are labeled with Rh-DOPE (*red*). The peptide interacts with the GUVs, disturbs their structure, and then enables the passage of fluorescent dyes by formation of pore-like structures. To see this figure in color, go online.

Milford, MA), adjusted to ~250 μL/min. During all experiments the source temperature was maintained at 80°C and the needle voltage at 3.6 kV, applying a drying gas flow (nitrogen) of 200 L/h and a nebulizer gas flow of 20 L/h. The mass spectrometer was calibrated with intact horse heart myoglobin and its typical cone-voltage-induced fragments. The molecular masses were determined by ESI mass spectrometry by adjusting the mass spectrometer to give a full width at half-maximum of 1 mass unit. The voltage between cone sample and skimmer lens that controlled the ion transfer to the mass analyzer was set to 38 V. Approximately 50 pmol (10 μL) of each sample was injected into the electrospray transport solvent. The ESI spectra were obtained in the multichannel acquisition mode; the mass spectrometer data acquisition and treatment system was equipped with MASSLYNX and TRANSFORM software (Waters) for handling spectra.

GUV formation

GUVs composed, respectively, of 100% DOPC (PC); 80% DOPC and 20% POPS (PC/PS); 90% DOPC and 10% DOPE (PC/PE); and 70% DOPC, 10% DOPE, and 20% POPS (PC/PE/PS) were formed by the electroformation method as follows. Chloroform stock solutions of the lipid mixture and the fluorescent lipid Rh-DOPE (0.5 mol %) were prepared at a total lipid concentration of 1.0 mM. A quantity of 50 μK of lipid solution was added dropwise onto the platinum wires of the electroformation chamber and dried under vacuum from 2 to 4 h. The chamber was then filled with a 300-mM sucrose solution and the peak-to-peak voltages of 6.5 V alternating current, for zwitterionic lipid mixtures, or 4.5 V alternating current, for anionic lipid mixtures, was applied across the platinum wires at distinct frequencies: 10 Hz for 30 min, 3.0 Hz for 15 min, 1.0 Hz for 7 min, and 0.5 Hz for 7 min. The GUVs were prepared at room temperature. Once formed, GUV solutions were harvested from the electroformation chamber and stored in foil-wrapped plastic vials before imaging.

LUV preparation

LUVs composed of the same lipid mixtures as the GUVs were prepared according to general procedures with slight modifications, as outlined below. Briefly, phospholipids dissolved in chloroform were dried under N_2 flow in a round bottom flask. The lipid film was completely dried under vacuum for at least 3 h and afterwards hydrated either with 10 mM phosphate buffer containing 150 mM NaF for binding experiments, or with ANTS 5 mM and DPX 8 mM both prepared in HEPES buffer for the requenching assay. In both preparations the final lipid concentration was ~10 mM. The suspension was submitted to two extrusion steps using an Avanti Mini-Extruder (Avanti Polar Lipids) and double-stacked polycarbonate membrane (Nucleopore Track-etch Membrane; Whatman, Florham Park, NJ): first, 6 times through 0.4 μm and then 11 times through 0.1 μm membranes. The dye-entrapped LUVs were separated from unencapsulated fluorophores by gel filtration on a Sephadex G25M column (Amersham Pharmacia Biotech, Piscataway, NJ). Vesicles were used within 48 h of preparation. The lipid concentrations were determined by phosphorus analysis (13).

CD spectroscopy for binding isotherms

A solution of 10 μM MP1 in buffer was titrated with increasing lipid concentrations of zwitterionic (PC and PC/PE) or anionic (PC/PS and PC/PE/PS) LUVs up to 1.5 mM. The highest lipid concentration used was $>10/K_p$, where K_p is the partitioning coefficient, which assures that all the peptides are bound in anionic and zwitterionic vesicles (14). After each lipid addition the CD spectra were collected from 260 to 190 nm, at 25°C, with a Jasco-815 spectropolarimeter (JASCO International, Tokyo, Japan), which was routinely calibrated at 290.5 nm using d-10-camphorsulfonic acid solution and a 0.2 cm path-length cell. The spectra were averaged over 15–30 scans, at a scan speed of 50 nm/min, bandwidth of 1.0 nm, 1.0 s response, and 0.2 nm resolution. After baseline correction, the observed ellipticity at 222 nm, θ (mdeg), was converted to mean residue ellipticity [Θ] (deg cm^2/dmol), using the relationship $[\Theta] = 100\theta/(l\ c\ N)$, where l is the path length in cm, c is the peptide concentration in mM, and N is the number of peptide residues.

The plots of the normalized molar ellipticity (Θ_{obs}/Θ_0) as a function of the lipid concentration were fitted with the equation

$$\frac{\Theta_{obs}}{\Theta_0} = 1 + \left(\frac{\Theta_H}{\Theta_0} - 1\right)\left(\frac{K_p[L]}{1 + K_p[L]}\right),$$

where Θ_0 is the molar ellipticity per residue at 222 nm in the absence of LUVs, $[L]$ is the total lipid concentration, and Θ_H/Θ_0 is the maximum-normalized molar ellipticity.

Confocal microscopy

Vesicle samples were imaged at room temperature by using a model No. LSM 510 META (Zeiss, Jena, Germany) inverted confocal microscope. The objective lens used was a 63×/1.4 N.A. Oil Plan-Apochromat. The Rhodamine probe was excited by a DPSS laser at 543 nm, the CF probe was excited with the 488 nm line of an Argon laser, the Cascade Blue was excited at 405 nm with a diode laser and the Alexa Fluor 647 was excited with the 633-nm line of a Helium Neon laser. Small plastic Petri dishes with glass coverslips on the bottom (Cat. No. P35G-1.5-14-C; MatTek, Ashland, MA) were used as observation chambers. The glass was treated with a 10% bovine serum albumin solution (10 min) and then washed with deionized water to avoid vesicle adhesion to the glass (15). A quantity of 20 μL of GUV solution and 75 μL of buffer were incubated for 15 min before imaging to allow the GUVs to sediment to the bottom of the sample. This is due to the higher density of the sucrose solution inside the GUVs in contrast to the surrounding buffered saline solution.

CF, 3k-CB, and 10k-AF647 were diluted in HEPES buffer to obtain solutions with final concentration of between of 3–30 μM and were pipetted carefully to the top of the sample. Images and movies of the vesicles in the mixture with buffer and the three dyes were recorded as a control experiment. For the quantitative analysis of the dye influx process, the concentration of dye in the interior of the vesicles was normalized on a scale of 0–1 according to the equation $c(t) = (b_{interior,\ t} - b_{interior,\ control})/(b_{exterior} - b_{interior,\ control})$. The value $b_{exterior}$ is the average pixel intensity in a region of the bulk solution, $b_{interior,\ t}$ is the average pixel intensity of dyes in the interior of GUVs after t seconds after peptide addition, and $b_{interior,\ control}$ is the average pixel intensity of the dyes in the interior of GUVs in the unleaked control samples (i.e., the background signal). This procedure allowed us to monitor leakage events of individual GUVs for a period of up to 30 min after peptide addition and to quantify the permeability of GUVs during leakage events.

Analysis of confocal images and movies

Confocal images and movies were analyzed using IMAGEJ software (National Institutes of Health, Bethesda, MD). As a criterion, aggregated and multilamellar vesicles and vesicles with a diameter <8 μm were excluded from the analysis. Images of GUVs were obtained after 30 min of MP1 addition at total final concentrations 4 or 40 nM, and 0.4, 1.2, or 4.0 μM. This was done to investigate the influx percentage of different-sized dyes as a function of peptide concentration (C_p). The percentage of dye influx was obtained by rescaling dye influx fraction c in terms of a percentage. In the movies, single vesicles were monitored over time immediately after peptide addition, at a fixed $C_p = 4.0$ μM, for a period of up to 30 min or until GUVs had fully leaked. For vesicles that moved during the course of the movie, the region of interest was changed to keep it within the vesicle interior. The average background intensity was also monitored over time by selecting an external region near the observed vesicle. Vesicle permeability

(P_m) was calculated as the gradient of the log-linear plot of the time-dependent influx, $-R/3 \ln(1 - c(t))$ against time t (16,17). R is the observed vesicle radius and $c(t)$ is the dye influx fraction.

ANTS/DPX requenching measurements

LUVs with entrapped 5 mM ANTS and 8 mM DPX in HEPES buffer, pH 7.4, were prepared and submitted to MP1 interaction to establish the mechanism of leakage for the four studied lipid compositions. It is possible by determining the dependence of the quenching of ANTS, with the quencher DPX, inside the vesicles (Q_{in}) as a function of the ANTS fraction outside the vesicles (f_{out}), to differentiate between all-or-none and gradual leakage mechanisms, as proposed by Ladokhin et al. (18). If Q_{in} remains constant and at ~0.2 as f_{out} increases, then the peptide exhibits an all-or-none mechanism of leakage. If Q_{in} increases with f_{out}, then it suggests the gradual mechanism of leakage. The ANTS fluorescence intensity was collected with a PC1 spectrofluorometer (ISS, Champaign, IL) with excitation at 355 nm and emission at 520 nm. Excitation and emission bandwidth were set at 1.0 and 2.0 nm, respectively. DPX 25 mM was titrated into samples containing 100 μM of lipid and increasing peptide concentrations ranging from 0 to 40 μM. Each peptide sample was kept 2 h to equilibrate until the fluorescence signal stabilized, to ensure that MP1 activity was complete. At the end, to obtain the fluorescence signal corresponding to the complete leakage, 25 μL of 10% Triton X-100 was added to each sample. The fluorescence signals were converted to Q_{in} and f_{out} and plotted to obtain the mechanism of leakage curve.

AFM

Lipids and cholesterol were purchased in dry form from Avanti Polar Lipids and solvated to 5 mM in chloroform. Supported bilayers were formed from these lipids by the vesicle fusion method. Specifically, solvated lipids were mixed in a glass vial to the correct molar proportion, dried under a gentle stream of N_2, and then placed under vacuum overnight to ensure no chloroform remained. The mixture was then hydrated using the buffer solution to a lipid concentration of ~0.5 mg/mL. The suspension was then tip-sonicated until the solution clarified, indicating formation of small unilamellar vesicles. The buffer solution used throughout bilayer preparation and imaging was 125 mM NaCl and 10 mM HEPES at pH 7.4.

A quantity of 100 μL of the lipid vesicle solution was pipetted onto a freshly cleaved mica substrate along with 50 μL of a solution of 10 mM $MgCl_2$ to aid vesicle fusion and create a perfect defect-free supported bilayer. The sample was then incubated in a humid environment at 50°C for ~1 h, allowing the vesicles to sediment and rupture on the surface to form a continuous bilayer. The bilayer was then rigorously rinsed 10 times with 100 μL warm (50°C) buffer using a pipette (Gilson, Madison, WI), the wash directed parallel to the bilayer surface to remove adhering vesicles.

AFM experiments were carried out using a Multimode 8 AFM on a Nanoscope V controller (Bruker, Billerica, MA), and equipped with a fluid cell. Bruker NP-A probes ($k \approx 0.35$ Nm^{-1}) were operated in contact mode. Although tapping mode in fluid often produces sharper images, it was found that the erosion of the bilayer by the antimicrobial peptide caused debris from the bilayer to float freely in solution, compromising the interactions between tip and sample. Contact mode is generally less susceptible to these effects and thus was more suited. It is also faster than peak-force tapping mode, important when attempting to capture dynamics. After acquisition of several images of the clean bilayer without peptide, the antimicrobial peptide was added manually to the bilayer using a pipette (Gilson). A quantity of 150 μM peptide in buffer was added such that the final peptide concentration within the cell was 4 or 10 μM. The MP1 concentration was chosen such that statistically significant membrane perturbations could be observed within the comparatively small imaging area of the AFM within an experimentally accessible timescale. Many different regions of each bilayer were imaged to ensure a representative view of the sample was obtained, unless time-resolved experiments were taking place, when the imaging region was fixed for the whole of one experiment to see the direct effect of peptide injection. Maximum scan size was equal to 13 μm. Each preparation of peptide on each lipid mixture was carried out at least three times, and usually five times, to assure reproducibility. Temperature was not controlled, but would stabilize between 25.5 and 26.0°C within 20 min of injection.

Phase contrast microscopy

GUVs were observed by phase contrast microscopy, in which the differences in density and refractive index between sucrose inside the vesicles and buffer outside the vesicles provide better contrast for observation under the microscope (model No. IX71, equipped with charge-coupled device cameras; Olympus, Melville, NY). Small plastic Petri dishes with glass coverslips on the bottom (Cat. No. P35G-1.5-14-C; MatTek) were used as observation chambers after a treatment with a 10% bovine serum albumin solution (10 min) (15). A quantity of 20 μL of GUV solution and 75 μL of buffer were incubated for 15 min before imaging to allow the GUVs to sediment to the bottom of the sample. After this, MP1 solution prepared in HEPES buffer was added to the observation chamber with a final concentration of 4 or 10 μM. The vesicles were observed under a 40× phase contrast objective for 2 h. The sizes of the GUVs before and during peptide interaction were obtained by measuring the average vesicle diameter for a period of 5 min at $t \approx 0$, 30, 60, 90, and 120 min.

RESULTS

PS lipids significantly enhance peptide binding to the membrane

The overall efficacy of a peptide at disrupting a target membrane can be broken down into the combination of two sequential steps: 1) binding of the peptide to the membrane surface, and 2) the efficiency of membrane disruption by the bound peptide resulting in membrane poration or leakage. First, we investigate the membrane binding isotherms of the MP1 peptide to our four lipid compositions of interest by CD spectroscopy by titrating a 10 μM MP1 solution with increasing lipid (LUV) concentrations (Fig. 2). Fitting these binding isotherms revealed that the partition coefficient (K_p) of the peptide was 7–8 times higher for membrane compositions containing PS (K_p values were PC 4600 M^{-1}, PC/PE 4000 M^{-1}, PC/PS 33,000 M^{-1}, and PC/PE/PS 30,000 M^{-1}). It is also interesting to note from this data that PE lipids slightly suppress peptide binding by a factor of ~10%. Due to the cationic nature of MP1 (net charge of $+2e$), it is highly likely that the enhanced peptide binding to anionic-PS-containing membranes is primarily driven by electrostatic interactions.

MP1 dose-response studies reveal that PE and PS lipids enhance membrane permeability at lower peptide concentrations

To investigate the efficiency of membrane disruption, we measured the leakage of macromolecules across GUV model membranes by confocal fluorescence microscopy. Fluorescent passive leakage markers of different sizes

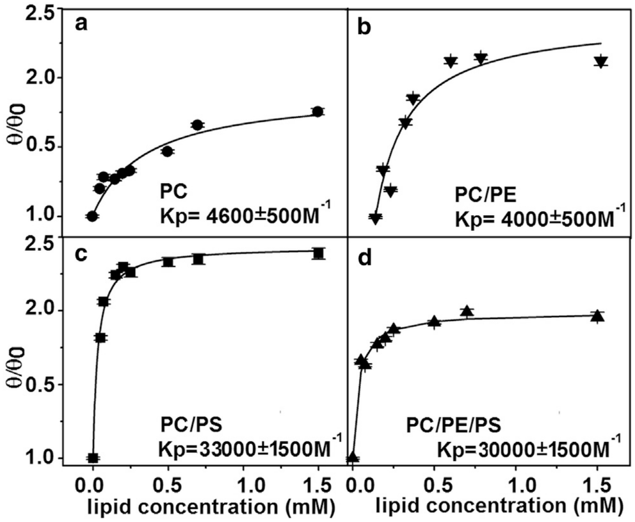

FIGURE 2 Binding isoterms show that MP1 has a higher affinity for PS-containing membranes. The binding isoterms and the partition coefficients (K_p) obtained using CD by lipid titration at 10 μM MP1 solution. LUVs are composed of (*a*) PC, (*b*) PC/PE, (*c*) PC/PS, and (*d*) PC/PE/PS.

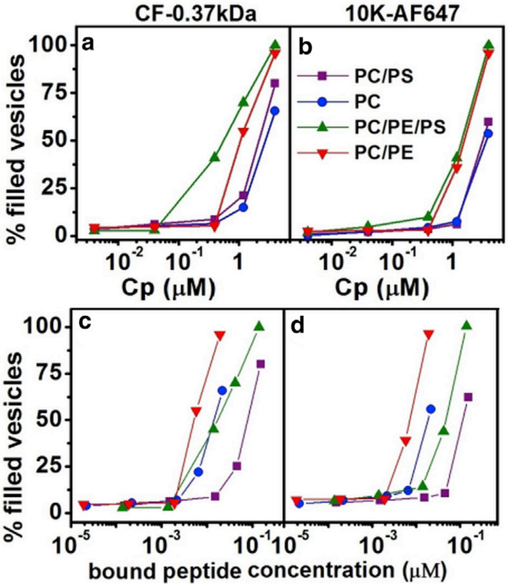

FIGURE 3 Dose-response curves show increased leakage of PE containing GUVs at lower peptide concentrations. (*a* and *c*) Percentage of GUVs filled by CF (0.37 kDa) after 30 min incubation time with MP1. (*b* and *d*) Percentage of GUVs filled by 10k-AF647 after 30 min incubation time with MP1. All vesicles presenting >20% of dye entry were accounted as filled. The data is plotted as a function of (*a* and *b*) total peptide concentration and (*c* and *d*) the concentration of peptide bound to the membranes. Fifty GUVs were used to construct each data point. Vesicles are composed of PC, PC/PS, PC/PE, and PC/PE/PS. To see this figure in color, go online.

were simultaneously employed: 0.37 kDa carboxyfluorescein (CF), 3 kDa dextran labeled with Cascade Blue (3k-CB), and 10 kDa dextran labeled with Alexa Fluor 647 (10k-AF647). GUVs were composed of PC, PC/PS, PC/PE, or PC/PE/PS. The dose-response of the membranes to the addition of MP1 was characterized for each membrane composition and passive leakage marker by evaluating the normalized fluorescence intensities of the probes in the intravesicular lumen of the GUVs after 30 min incubation time (Fig. 3 and Fig. S1 in the Supporting Material). Each data point in Figs. 3 and S1 shows the mean leakage of 50 individual GUVs from a minimum of two independent experiments. For determining the percentage of leaked vesicles (Fig. 3), a threshold of 20% leakage (normalized to the background probe concentration) was used to define a filled vesicle. Alternatively, this data can be analyzed in terms of the average leakage into GUVs as a percentage of the probe concentration in the external medium (Fig. S1).

The integrity of membranes containing both PE and PS lipids is perturbed by lower concentrations of MP1 peptide than the other membrane compositions we investigated. PC/PE/PS GUVs show significant (40–65%) leakage to the CF probe at 0.4 and 1.2 μM MP1 concentrations, whereas other membrane compositions studied leaked <30% within this concentration range (Fig. S1 *a*).

Larger pore defects, evidenced by leakage of the larger 10k-AF647 probe, are shown to be significantly enhanced in membranes containing 10% PE. Almost all GUVs (98%) containing PE lipids are observed to leak the 10k-AF647 probe when in the presence of 4.0 μM MP1, compared to <60% of GUVs for other membrane compositions at the same peptide concentration (Fig. 3 *b*). This is the most significant enhancement in selective perturbation for specific lipid membrane compositions observed within the dose-response data in Figs. 3 and S1. At this MP1 concentration, membranes under native conditions would be susceptible to the leakage of biological macromolecules such as small proteins and RNAs.

Interestingly, we also plot the GUV leakage data as a function of the concentration of bound peptide on the membrane using the specific partition coefficients of the peptide for different lipid compositions that were calculated in Fig. 2 (see also Figs. 3, *c* and *d*, and S1, *c* and *d*). This representation of the data clearly shows that PE lipids increase the susceptibility of PC membranes to disruption by the MP1 peptide, with PC/PE lipids leaking at significantly lower bound peptide concentrations. Due to the higher bound concentration of peptide to membranes containing PS lipids, this lipid decreases the apparent susceptibility of the membrane to leakage as observed by the onset of leakage shifting to higher bound peptide concentrations. For PC/PE/PS GUVs, the apparent competing effects of PE and PS lipids on the membrane's leakage susceptibility roughly cancel each other out, leading to intermediate membrane disruption susceptibility for a given bound peptide concentration. However, the effect of increased bound peptide concentrations due to PS far outweighs its apparent inhibition of membrane leakage, making PC/PE/PS GUVs the most susceptible to leakage for a given total peptide concentration. Therefore, the combined roles of PS in increasing

membrane binding and PE in increasing the susceptibility of the membrane are both important in increasing the membrane disruptive efficacy of MP1.

Confirmation of the pore-formation hypothesis in lipid vesicles

Fluorescence spectroscopy experiments using LUVs give ensemble-averaged measurements with high statistics on a large population of vesicles, complementing single-vesicle GUV imaging experiments that inherently have lower statistics but yield information on the distribution of behaviors and rare events within a sample. The fluorescence requenching method (18) enables us to distinguish the type of leakage mechanism induced by MP1 for the lipid compositions under investigation. One possibility is the all-or-none mechanism where some vesicles release all of their internal contents while the others remain intact. This is attributed to pore-formation mechanisms of membrane perturbation, or complete vesicle lysis. Another possibility is the gradual leakage mechanism where vesicles only release a fraction of their encapsulated contents during a leakage event. This is associated with transient perturbations of the membrane. A fluorophore (ANTS) and a quencher (DPX) are encapsulated within lipid vesicles at high concentrations such that the fluorescence is initially quenched; vesicle leakage results in the release of both ANTS and DPX, but quenching is decreased due to dilution of these probes. The externalized ANTS fluorescence can be suppressed by additional titration of DPX such that the remaining fluorescence signal is only due to the ANTS inside intact vesicles. The data can be represented by a plot of the degree of quenching (Q_{in}) against the released ANTS fraction (f_{out}). In the case of an all-or-none leakage mechanism, the plot of Q_{in} versus f_{out} will show no dependence of Q_{in} on f_{out}. In contrast, the gradual leakage mechanism causes release of only a fraction of the encapsulated contents within individual vesicles and so Q_{in} increases with increasing f_{out} (18).

Fig. 4 shows that the values of Q_{in} remain constant with the increase of f_{out} and the consequent increase of peptide/lipid molar ratios. This clearly shows that MP1 exhibits the all-or-none leakage mechanism for all lipid compositions studied, which is in contrast to what has been observed for antimicrobial peptides mastoparan X and mastoparan MP (19). We propose that this all-or-none leakage is related to peptide-induced pore formation (20–23), where the vesicles are able to release all their internal contents through pore-like structures that are sufficiently long lived (23–28). We do not solely attribute the all-or-none leakage to lysis of the vesicles because nonlysed, leaky vesicles are observed in our GUV experiments (Figs. 3 and S1). However, we do not discount the possibility that lysis might play a role in the LUV leakage at the highest peptide concentrations used in this assay. Furthermore, pore-like activity of MP1 has previously been identified from electrophysiology measurements in planar lipid bilayers composed of phytanoyl-PC and phytanoyl-PC/PS (70:30) (4).

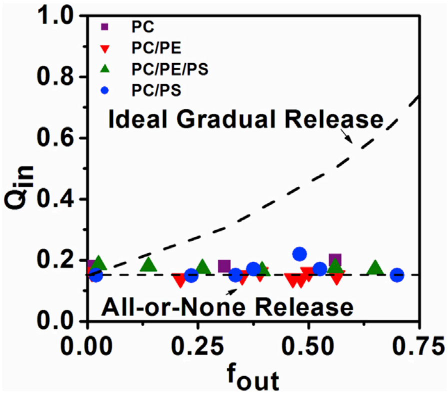

FIGURE 4 Fluorescence requenching assays for MP1 reveal all-or-none leakage in the four lipid compositions studied. Q_{in} is constant as a function of f_{out} for MP1, which is in agreement with the all-or-none mechanism of dye release.) (*Lines*) Theoretical curves for ideal graded and all-or-none dye release (18). To see this figure in color, go online.

Our fluorescence requenching results show that stable pores form with a lifetime that persists long enough for the dye efflux to reach equilibrium in LUV systems. However, this does not discount the possibility that pores might be transient over longer timescales, for example during the leakage of much larger vesicles such as GUVs where the encapsulated volume of dye that needs to be released during a leakage event is ~10^6 times greater than for the LUV model system. Indeed, we will see some evidence for transient pore events and dynamic changes in membrane permeability in the single GUV leakage kinetics data that follows. Nevertheless, all-or-none leakage is clearly evident in GUVs after 30 min incubation with 1.2 and 4.0 μM MP1. Leakage histograms of the individual GUVs (an alternative representation of data shown in Figs. 3 and S1) predominantly show either $\lesssim$20% (unleaked) or $\gtrsim$80% (fully leaked) leakage (Fig. S2).

Synergistic enhancement of GUV leakage kinetics by PE and PS lipids

Analysis of the time delay from the start of our GUV experiments (addition of the peptide) to observations of the onset of GUV leakage reveals a synergistic reduction in this lag time for PC/PE/PS membranes (Table 1 and Fig. 5). In these GUV experiments, we add 4.0 μM MP1 to our samples and monitor the time taken for initial leakage events of GUVs to the 0.37 kDa CF probe to occur ($t_0 - t_{CF}$). This MP1 concentration is chosen as it causes significant leakage of GUVs within 30 min of peptide addition across all four lipid compositions of interest. The onset of leakage occurs approximately twice as quickly for PC/PE/PS GUVs than for other membrane compositions, with only a very slight reduction of the lag time for PC/PS membranes compared

TABLE 1 Lag times between the onset of leakage of each dye and the time interval before the initial leakage takes place after the addition of peptide

Time Delays (s)	PC/PE	PC/PE/PS	PC/PS	PC
$t_0 - t_{CF}$	1600 ± 110	760 ± 120	1400 ± 60	1600
$t_{CF} - t_{3k\text{-}CB}$	1.8 ± 0.6	1.5 ± 0.3	41 ± 5[a]	160 ± 110
$t_{CF} - t_{10k\text{-}AF647}$	4.2 ± 1.5	2.0 ± 0.4	52 ± 6	220 ± 66
$t_{3k\text{-}CB} - t_{10k\text{-}AF647}$	2.7 ± 0.9	1.4 ± 0.4	9.6 ± 1.2	60 ± 42

The errors represent the standard deviation of the observed GUV data set.
[a]For PC/PS GUVs, the $t_{CF} - t_{3k\text{-}CB}$ data only includes GUVs that leaked to all three dyes; these sample conditions contained two distinct populations of GUV leakage behaviors where a second population of GUVs only leaked to the CF and 3k-CB dyes with a time delay of $t_{CF} - t_{3k\text{-}CB} = 4.8 \pm 0.6$ s.

with PC/PE and PC GUVs. Therefore, this is not a purely electrostatic effect from the increased rate and extent of peptide binding to anionic PS-containing membranes; it also requires the presence of PE to significantly increase the susceptibility of the membrane to permeabilization.

We also quantify the average delay times between leakage of the different-sized fluorescent probes between CF and 3k-CB ($t_{CF} - t_{3k\text{-}CB}$), CF and 10k-AF647 ($t_{CF} - t_{10k\text{-}AF647}$), and 3k-CB and 10k-AF647 ($t_{3k\text{-}CB} - t_{10k\text{-}AF647}$). Once the initial leakage event occurs, PE-containing GUVs rapidly become leaky to fluorescent probes of larger sizes (3 and 10 kDa). For PC/PE/PS and PC/PE membranes, GUVs become leaky to larger 3k-CB, then 10k-AF647 passive leakage markers within seconds of permeabilization to the smallest CF (0.37 kDa) probe (Table 1). The consecutive delay times between CF and 3k-CB probes and 3k-CB and 10k-AF647 probes were approximately an order-of-magnitude longer for PC/PS membranes, and almost two orders-of-magnitude longer for purely PC membranes. This strongly implies that the presence of PE significantly enhances the favorability and rate of formation of larger membrane defects or pores.

PE lipids significantly enhance pore size and membrane permeability

We use time-series confocal microscopy imaging to quantify the membrane permeability of GUVs during initial leakage. Quantification of the fluorescence intensity of the leakage markers in the intravesicular and extravesicular medium allows us to calculate the fractional leakage of individual GUVs as a function of time. The leakage kinetics of individual GUVs are monitored for up to 30 min after the addition of 4.0 μM MP1. This concentration is chosen as all lipid compositions show significant leakage within 30 min; a higher MP1 concentration of 10 μM is observed to induce significant lysis of GUV samples (Fig. S3). These experiments are conducted on GUVs of all four membrane compositions under investigation, using the CF, 3k-CB, and 10k-AF647 leakage markers simultaneously. This allows the time evolution of membrane permeability

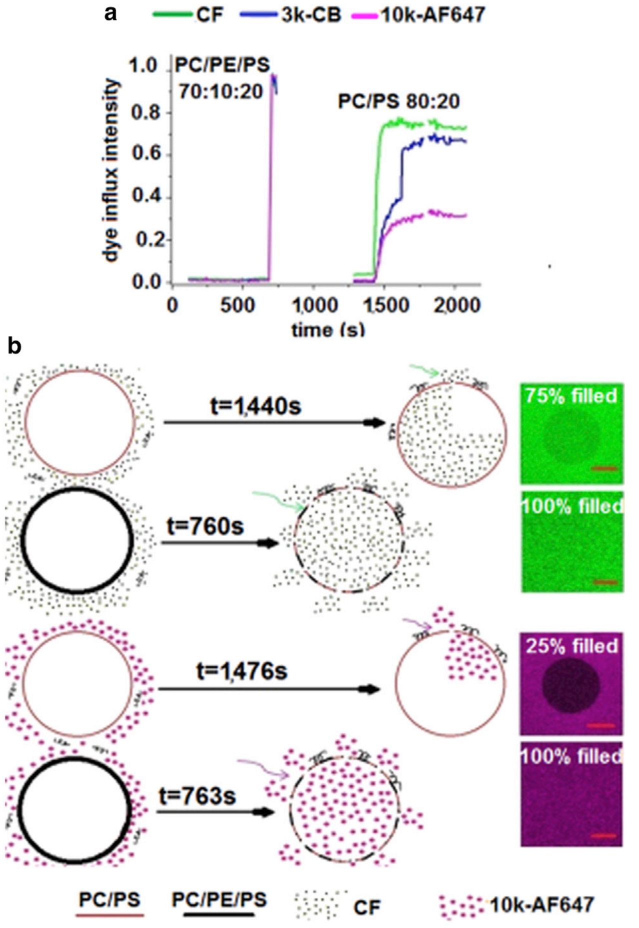

FIGURE 5 GUV permeabilization kinetics are synergistically enhanced by PE and PS lipids. (*a*) Comparison between dye influx kinetics of three distinct dyes (CF-0.37kDa, 3k-CB, and 10k-AF647) for PC/PE/PS and PC/PS GUVs. The time axis represents the time after peptide addition. These individual GUV leakage profiles were chosen as they represent the average behavior of the GUVs observed under these conditions. (*b*) Schematic representation of the dye influx kinetics for PC/PS and PC/PE/PS GUVs in the presence of CF and 10k-AF647 passive leakage markers. This shows the average lag times for GUV leakage after the addition of 4.0 μM MP1 and the typical average leakage extent of the GUVs that resulted in these experiments. To see this figure in color, go online.

to different molecular sizes to be simultaneously measured for individual GUVs (Figs. 5 *a* and S4). To the best of our knowledge, this is the first example of simultaneous size-dependent permeability measurements in GUVs for three different-sized leakage markers.

Typical leakage kinetic profiles for different membrane compositions and probe sizes are shown in Figs. S4 and 5 *a*. It can be qualitatively seen from these example profiles that membrane compositions containing 10% PE exhibit full and rapid membrane leakage for all three sizes of leakage marker, consistent with the leakage kinetics data in Table 1, which is also outlined in Fig. 5 *b*. For membrane compositions lacking PE, the leakage rates can sometimes be seen to increase and decrease intermittently, sometime plateauing before full leakage is achieved; this

is particularly evident in the leakage profile of a single PC/PS GUV shown in Fig. S5. We attribute these observations to membrane self-healing events, where the pores/defects reseal and the membrane regains its permeability barrier, followed by later phases of increased leakage. This is particularly observed for the larger 3k-CB and 10k-AF647 leakage probes. Therefore, the membrane permeability for PC and PC/PS GUVs, in particular, can change dynamically during the observed leakage events; this is a result of the competition between the lipid bilayer and peptides in maintaining their barrier properties and inducing membrane pores, respectively (Fig. S5).

Our leakage kinetic profiles were used to calculate the membrane permeability to the different-sized probes using a diffusional model for membrane translocation (29); the membrane permeability is the gradient of the log-linear plot as seen in the example data in Fig. 6, *a* and *c*. Average permeability values for each membrane composition to each probe size during the initial leakage events are shown in Table 2. It can be seen that, for all membrane compositions tested, average permeability decreases with increasing probe size. However, the most significant finding from this data is the large, one-order-of-magnitude increase, in membrane permeability for membrane compositions containing 10% PE. This can be observed for all three leakage markers

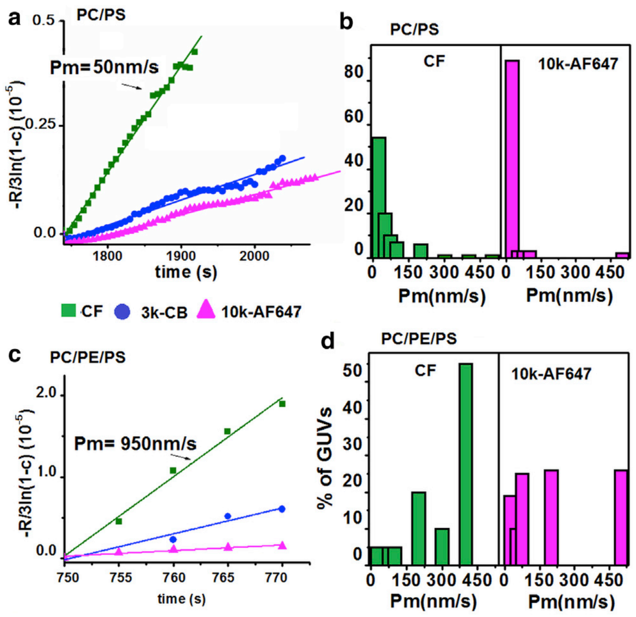

FIGURE 6 PE lipids facilitate much greater membrane permeability in GUV membranes. (*a*) Typical log-linear plot of time-dependent dye influx: $-R/3\ln(1-c)$ (10$^-$5) versus time, for the three dyes in a single GUV of PC/PS. (*b*) Distributions of the obtained permeabilities in single GUVs composed of PC/PS. (*c*) Typical log-linear plot of time-dependent dye influx: $-R/3\ln(1-c)$ (10^{-5}) versus time, for the three dyes in a single GUV of PC/PE/PS. (*d*) Distributions of the obtained permeabilities in single GUVs composed of PC/PE/PS. The permeabilities are obtained from the slopes of the log-linear plots of the time-dependent influx of dyes into single GUVs. To see this figure in color, go online.

studied. It can also be seen that the presence of PS in the membrane imparts a modest, but significant, increase in permeability on the membranes upon perturbation by MP1. This effect can be seen further in Fig. 6, *b* and *d*, which show the distributions of permeability measurements for PC/PS and PC/PE/PS GUVs to the CF and 10k-AF647 leakage markers, respectively. For both probe sizes, the majority of permeability measurements for PC/PS membranes were in the 0–25 nm/s range, whereas when PE was included in the membrane formulations, a large proportion of permeability measurements were >500 nm/s.

It should be noted that the observed permeability distributions (Fig. 6, *b* and *d*) are broad due to the fact that peptide-induced pores do not have well-defined structures, pore formation events are stochastic, and the membrane interfaces are fluid, giving rise to this wide distribution of individual permeability events when measured at the single vesicle level. Indeed, it has previously been reported that the initial pores that form during peptide-induced pore formation might be far from equilibrium and can, for example, relax to a smaller size over longer timescales as has been observed for the peptides Bax-α5 (23) and magainin 2 (30).

The permeability data was used to calculate the effective fractional permeable area of the membrane for each probe size using the expression (29)

$$\frac{A_p}{A_v} = \frac{P_m}{D_0}\delta,$$

where A_p is the permeable area of membrane on a GUV, A_v is the total area of the vesicle, P_m is the permeability, and δ is the thickness of the membrane. The Stokes-Einstein diffusion constant of the leakage marker is $D_0 = kT/6\pi\eta R_0$, where kT is the thermal energy, η is the solvent viscosity, and R_0 is the hydrodynamic radius of the fluorescent dye that was estimated with the relation $R_0 = 0.0332(M_w)^{0.463}$ in nanometers (31); M_w is the molecular weight of the dye. A brief derivation of this equation is presented in the Supporting Material. It should be noted that this equation is most accurate for the formation of large membrane pores as it assumes that the diffusion constant of the dye within the pore is the same as its diffusion constant in bulk solution. However, we believe this to be a reasonable assumption because these passive leakage markers will have a very short residence time within the pore itself due to the bilayer only being ~5-nm thick; these solutes are not expected to interact strongly with the membrane itself.

Values of the fractional permeable areas are shown in Table 2. The fractional permeable areas were also found to be an order-of-magnitude greater for membrane compositions containing PE than for those that did not. Note that slightly larger permeable areas were measured for the larger leakage markers; these represent a later time point in the membrane disruption of GUVs by MP1 as the smaller leakage markers translocate the membrane at earlier times (Table 1). This extended delay time therefore allows for a

TABLE 2 Average permeability values ($\langle P_m \rangle$) and the average fractional permeated area per vesicle ($\langle A_p \rangle / \langle A_v \rangle$) obtained from the average permeability values for each probe size and membrane composition

	$\langle P_m \rangle_{CF}$ (nm/s)	$\langle A_p \rangle / \langle A_v \rangle_{CF}$ (10^{-6})	$\langle P_m \rangle_{3k\text{-}CB}$ (nm/s)	$\langle A_p \rangle / \langle A_v \rangle_{3k\text{-}CB}$ (10^{-6})	$\langle P_m \rangle_{10k\text{-}AF647}$ (nm/s)	$\langle A_p \rangle / \langle A_v \rangle_{10k\text{-}AF647}$ (10^{-6})
PC	46 ± 14	0.45 ± 0.14	17 ± 6	0.50 ± 0.16	8 ± 2	0.44 ± 0.08
PC/PS	59 ± 12	0.58 ± 0.12	29 ± 6	0.90 ± 0.20	23 ± 4	1.30 ± 0.20
PC/PE	466 ± 143	4.60 ± 1.40	207 ± 102	6.50 ± 3.40	158 ± 53	8.80 ± 2.90
PC/PE/PS	589 ± 142	5.80 ± 1.40	333 ± 73	10.40 ± 2.80	169 ± 52	9.40 ± 2.90

The errors represent the standard deviation of the observed GUV data set.

greater area of membrane disruption to occur before the initiation of leakage to the larger M_w dyes.

Besides the order-of-magnitude increase in membrane permeabilization in the presence of PE lipids, we found an interesting correlation between PE content and membrane morphological response to MP1. Without PE, PC and PC/PS GUVs exhibited bright spots of fluorescent lipids at specific locations on the membrane surface in the presence of 4.0 μM MP1 (Fig. 7). We attribute these observations to local aggregation of peptides and lipids at the GUV surface. These peptide-induced lipid aggregates may be in competition with the pore-/defect-forming activity of the peptides. Such dense lipid structures were not seen on GUVs containing PE (PC/PE and PC/PE/PS) upon introduction of the peptide. Therefore, we speculate that the PE suppresses the intramembrane lipid aggregation by more easily facilitating the poration of the membrane.

While localized lipid aggregation was not observed on the surface of the PE-containing GUVs, these GUVs were observed to decrease in diameter by ~10–15% over a period of ~1 h after peptide addition (Fig. S6). Contrary to this, PC and PC/PS GUVs remained at a constant size for up to 2 h after addition of MP1. Therefore, MP1 results in the significant loss of lipid from only those GUVs that contain PE lipids.

Direct imaging of peptide-induced pores by AFM

AFM imaging of supported lipid bilayers confirms the role of PE in potentiating the formation of larger transmembrane pores. MP1 was added at 10 μM concentration to induce significant pore formation on the relatively small patches of membrane imaged by the AFM within a reasonable experimental timescale (<2 h); resultant pores/defects were observed to be much larger in PC/PE/PS membranes (250 ± 110 nm in diameter) compared to PC/PS (54 ± 30 nm in diameter) (Fig. 8). Similar-sized transmembrane pores were observed in PC/PE/PS and PC/PE membranes (290 ± 200 nm in diameter), but significantly fewer defects formed in PC/PE membranes. Note that the large standard deviations in these average pore diameters represent a significant size polydispersity in the defects formed. No pores were evident in PC membranes 2 h after peptide addition (Fig. S7); however, pores would need to be several nanometers in diameter to be observable by AFM, considerably larger than those that can be detected by passive dye influx into the GUVs we used to investigate the early stages of GUV poration.

Our AFM studies also clearly show a difference in pore formation and growth mechanisms dependent on the presence of PE. The large transmembrane pores in PC/PE/PS and PC/PE membranes are seen to grow by the stepwise loss of lipid aggregates from the edge of the pore, implying that vesicle micellization is important for pore growth in the these membranes (Fig. S8). This is consistent with the small decrease (within experimental error) in GUV size observed for PE-containing GUVs by phase contrast microscopy (Fig. S6). Conversely, in PC/PS membranes, raised areas of lipid are first seen to form on the membrane (Fig. S9), which may correlate to the dense lipid structures observed in Fig. 7. These raised areas of membrane later evolve into comparatively small pores; many of the defects seen in Fig. 8 (*bottom left*) only span half the bilayer, with only the center of a few of these defects showing full bilayer pores (Fig. S10). This indicates that pores in these membranes may form via a half-membrane intermediate state. Finally the timescale for observation of membrane defects by AFM was much faster for PC/PE/PS membranes than

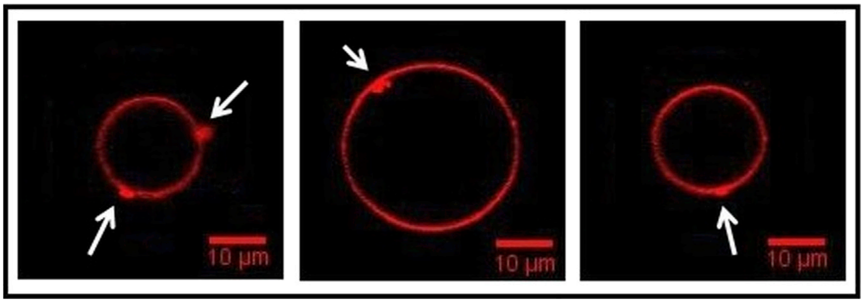

FIGURE 7 Lipid aggregation is observed within the membranes of GUVs lacking in PE. Images of local lipid aggregation at the GUV surface (bright localized spots of fluorescence) seen after peptide addition ($C_p = 4.0$ μM). This effect is frequently observed in PC and PC/PS GUVs, but not for the lipid mixtures containing 10 mol % PE. To see this figure in color, go online.

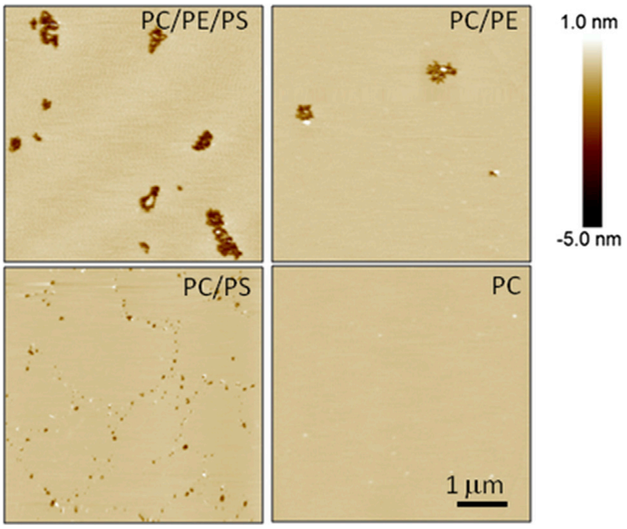

FIGURE 8 AFM images provide direct evidence that larger pores form in PE-containing membranes. AFM images of the four different membrane compositions 2 h after the addition of 10 μM MP1. Large pores can clearly be seen in PC/PE/PS and PC/PE membranes with a larger number of pores in the former. PC/PS shows much smaller defects, while no membrane perturbation is resolvable by AFM for PC membranes. To see this figure in color, go online.

for other lipid mixtures, with defects observed almost immediately after peptide addition (Fig S7), compared with a few tens of minutes for other mixtures.

DISCUSSION

Biophysical implications for MP1-lipid membrane interactions

We have shown a synergistic enhancement of the rate and extent of membrane permeabilization by MP1 peptides when PE and PS lipids are present in the lipid membrane. This picture is confirmed and corroborated by complementary experiments using three different model membrane systems: LUVs, GUVs, and planar-supported bilayers. We consider the perturbation of the membrane by MP1 peptides in two steps: 1) binding of the peptides to the membrane, and 2) perturbation of the bilayer structure by bound peptides to induce leakage.

Binding isotherms (Fig. 2) reveal that PS lipids cause a 7–8-fold increase in peptide bound to the membrane. This strongly outweighs the small ~10% reduction in bound peptide concentration caused by the PE lipids. Therefore, we find that the dominant role of PS lipids' contribution to the membrane disruption by MP1 is a large increase in peptide binding to the membrane.

The role of PE lipids in MP1-induced membrane disruption is twofold: 1) PE increases the susceptibility of the membrane to permeabilization by bound peptides, and 2) PE facilitates the formation of larger transmembrane pores. First, when the extent of GUV leakage is normalized to bound peptide concentration in the dose-response curves in Fig. 3, *c* and *d*, it can be seen that 4–5 times lower bound peptide concentration is required to induce a similar leakage response compared to comparable GUVs without PE lipids. Second, GUV and AFM experiments corroborate the effect of larger pores forming in the presence of PE. Quantitative analysis of GUV leakage profiles in Fig. 6 and Table 2 reveal that the presence of PE increases the permeability of membranes by an order of magnitude compared to membranes lacking in PE. Furthermore, once pores formed in GUVs, they quickly (within seconds) grew large enough in size to allow larger macromolecules (3 and 10 kDa) to permeate the membrane (Table 1); this compared to several tens of seconds for larger pores to form in GUVs lacking PE. Crucially, the formation of larger pores for PE-containing membranes is directly visualized by AFM (Fig. 8), where the observed pore diameters are ~5 times larger in the presence of PE (and hence ~20–30 times larger in average pore area, consistent with the order-of-magnitude increase in permeability reported for the GUVs).

The formation of transmembrane pores was confirmed by complementary experimental systems and techniques. Rapid translocation of membrane-impermeable leakage markers across GUV membranes, an all-or-none LUV fluorescence leakage assay, and direct visualization of transmembrane defects by AFM imaging of planar bilayers, all confirm this to be true. While these pores are fairly long lived, the membranes were sometimes observed to temporarily reseal, regaining their barrier properties. This can clearly be seen in the leakage profiles of individual GUVs in Figs. 5 *a*, S4, and S5. GUV and planar bilayer imaging experiments also strongly suggest differences in the mechanism of pore formation depending on whether PE lipids are present. Images of GUVs that did not contain PE lipids often exhibited bright spots of increased local lipid concentrations on the membrane, which we interpret to be local aggregation of peptides and lipid (Fig. 7). Similarly, AFM images showed locally raised regions of lipid scattered across the membrane for these lipid compositions (Fig. S9) before the formation of pores (Fig. S10). This contrasted to the pore-formation mechanism observed in the presence of PE, where local aggregates were not directly observed on the GUV surface and time-resolved AFM imaging showed pore growth to occur by the stepwise micellization and loss of lipid from the edge of the pores (Fig. S8).

Besides the increased binding due to PS and the increased membrane susceptibility and pore size due to PE, the synergistic enhancement of membrane disruption facilitated by these lipids can be observed in the kinetics of initial permeabilization events. GUV experiments showed that PC/PE/PS GUVs leaked a factor-of-two quicker than other membrane compositions (Table 1). This is again corroborated by the AFM studies where defects were observed in PC/PE/PS membranes almost immediately after peptide addition,

whereas perturbations of other membrane compositions took a few 10 s of minutes to evolve. The complementary pore-promoting effects of PS on bound peptide concentrations and PE on membrane susceptibility far outweigh their slight inhibitory effects on each other's roles (PE causes a slight reduction in binding affinity (Fig. 2) and PS causes a decrease in the membrane susceptibility to bound peptide (Fig. 3, *c* and *d*)). This is apparent from the effects of MP1 on GUVs, where PC/PE/PS membranes experience the greatest membrane perturbation for any given total peptide concentration (Fig. 3, *a* and *b*) and the larger number of pores observed on the membrane surface by AFM (Fig. 8). Therefore, our combined results provide a detailed mechanistic picture of the importance of increased PS and PE lipid concentrations in synergistically enhancing the membrane's propensity for significant disruption of its barrier properties by MP1 peptides.

Variations in lipid composition are responsible for differences in membrane properties such as charge, fluidity, lateral pressure profiles, and mechanical moduli. Changes in these fundamental membrane properties directly affect their interactions with extraneous compounds, such as antimicrobial peptides. The cationic nature of the MP1 peptide is likely an important component in the initial step of peptide action, in which the peptide recognizes the target membrane due to electrostatic interactions and binds to it in a structured form, most of the time as a helix. Therefore, the inclusion of anionic PS lipids in these membranes increases these electrostatic interactions with the MP1 peptide (net charge $= +2e$). However, MP1-membrane interactions cannot be solely driven by electrostatics as these peptides were also found to disrupt zwitterionic PC and PC/PE membranes, likely through secondary hydrophobic initial binding interactions that lead to a significantly lower bound concentration of peptide compared to the anionic membranes.

Next, insertion of the peptide into the bilayer likely takes place due to the hydrophobic effect, where nonpolar MP1 residues insert into the bilayer core, and defects may then be opened within the membrane structure, leading to its disruption. Furthermore, taking account of the fact that MP1 is a short peptide (14 amino acids) and hence not long enough to form a bilayer-spanning barrel stave pore (9,32), we anticipate that these pores will be disorganized toroidal structures formed by lipids and peptides, as described by many molecular-dynamics studies (33,34). PE is known to significantly modulate the lateral pressure profile through membranes and thereby induce negative curvature stress in the bilayer. Negative curvature stress has been shown to enhance the formation of toroidal lipid pores within a membrane by stabilizing the curvature of these structures (35). Therefore, PE would be expected to favor the formation of pore-like defects in the membrane, consistent with the increase susceptibility of these membranes to MP1-induced poration and the order-of-magnitude increase in membrane permeability that we find for PE-containing membranes upon interaction with MP1 peptides.

Implications for the chemotherapeutic potential of MP1 peptides

The MP1 peptide has been shown to have selective inhibition against numerous cancer lines compared to healthy cells (2,3). Such malignant cells are also known to have increased expression of PS and PE lipids on their outer plasma membrane (5–7). This study strongly correlates the enhanced tumor inhibitory effects of these peptides with this pathological change in plasma membrane lipid composition, where the upregulation of PS and PE lipids can synergistically enhance the membrane-permeabilizing activity of MP1. This membrane permeabilization is likely to be the primary mechanism of cancer cell death induced by these peptides.

This suggests that MP1 might be a candidate therapeutic for development of novel cancer therapies, or at least guide the development of novel lead compounds for treatment of these diseases. One challenge for the application of antimicrobial peptides in medicine is that they often do not show high enough selectivity to their target cells to result in a favorable therapeutic index for these compounds (36). However, MP1 does not exhibit hemolytic activity to rat erythrocytes but presents chemotaxis for polymorphonucleated leukocytes and potent antimicrobial action against Gram-positive and Gram-negative bacteria (12), suggesting it could have favorable selectivity. It may also be of interest to test MP1 in a combination therapy with other chemotherapeutics that have intracellular targets. The selectivity of the MP1 peptide to disrupt the membranes of cancer cells may act synergistically with these other drugs to significantly enhance the therapeutic potency. Therefore, the therapeutic potential of this and other membrane-active peptides within the field of oncology is worthy of further investigation.

SUPPORTING MATERIAL

Supporting Materials and Methods and ten figures are available at http://www.biophysj.org/biophysj/supplemental/S0006-3495(15)00768-7.

AUTHOR CONTRIBUTIONS

P.A.B., J.R.N., and S.D.C. designed the research; N.B.L. and A.A.-R. performed the research; M.S.P. provided new reagents; N.B.L., A.A.-R., S.D.C., J.R.N., and P.A.B. analyzed the data; and N.B.L., S.D.C., J.R.N., and P.A.B. wrote the article.

ACKNOWLEDGMENTS

P.A.B. acknowledges funding and support from the Biomedical & Health Research Centre at the University of Leeds and European Union Marie Curie Career Integration Grant BioNanoMuTT (No. PCIG09-GA-2011-293643).

J.R.N. and M.S.P. acknowledge financial support from the Sao Paulo Research Foundation (Fundação de Amparo à Pesquisa do Estado de São Paulo) under grants No. 2011/11640-5 and No. 2011/51684-1. J.R.N. and M.S.P. are researchers for Brazil's National Council for Scientific and Technological Development. N.B.L. has a Ph.D. scholarship from the Coordenação de Aperfeiçoamento de Pessoal de Nível Superior. A.A.-R. and S.D.C. acknowledge funding from the Engineering and Physical Sciences Research Council program grant CAPITALS (No. EP/J017566/1).

REFERENCES

1. de Souza, B. M., A. V. da Silva, …, M. S. Palma. 2009. Characterization of two novel polyfunctional mastoparan peptides from the venom of the social wasp *Polybia paulista*. *Peptides.* 30:1387–1395.
2. Wang, K. R., B. Z. Zhang, …, R. Wang. 2008. Antitumor effects, cell selectivity and structure-activity relationship of a novel antimicrobial peptide polybia-MPI. *Peptides.* 29:963–968.
3. Wang, K. R., J. X. Yan, …, R. Wang. 2009. Novel mode of action of polybia-MPI, a novel antimicrobial peptide, in multi-drug resistant leukemic cells. *Cancer Lett.* 278:65–72.
4. dos Santos Cabrera, M. P., M. Arcisio-Miranda, …, M. S. Palma. 2012. Influence of the bilayer composition on the binding and membrane disrupting effect of Polybia-MP1, an antimicrobial mastoparan peptide with leukemic T-lymphocyte cell selectivity. *Biochemistry.* 51:4898–4908.
5. Zwaal, R. F. A., P. Comfurius, and E. M. Bevers. 2005. Surface exposure of phosphatidylserine in pathological cells. *Cell. Mol. Life Sci.* 62:971–988.
6. Schweizer, F. 2009. Cationic amphiphilic peptides with cancer-selective toxicity. *Eur. J. Pharmacol.* 625:190–194.
7. Stafford, J. H., and P. E. Thorpe. 2011. Increased exposure of phosphatidylethanolamine on the surface of tumor vascular endothelium. *Neoplasia.* 13:299–308.
8. Wimley, W. C., and K. Hristova. 2011. Antimicrobial peptides: successes, challenges and unanswered questions. *J. Membr. Biol.* 239:27–34.
9. Pasupuleti, M., A. Schmidtchen, and M. Malmsten. 2012. Antimicrobial peptides: key components of the innate immune system. *Crit. Rev. Biotechnol.* 32:143–171.
10. Fjell, C. D., J. A. Hiss, …, G. Schneider. 2012. Designing antimicrobial peptides: form follows function. *Nat. Rev. Drug Discov.* 11:37–51.
11. Rakowska, P. D., H. Jiang, …, M. G. Ryadnov. 2013. Nanoscale imaging reveals laterally expanding antimicrobial pores in lipid bilayers. *Proc. Natl. Acad. Sci. USA.* 110:8918–8923.
12. de Souza, B. M., M. A. Mendes, …, M. S. Palma. 2005. Structural and functional characterization of two novel peptide toxins isolated from the venom of the social wasp *Polybia paulista*. *Peptides.* 26:2157–2164.
13. Rouser, G., S. Fkeischer, and A. Yamamoto. 1970. Two-dimensional thin layer chromatographic separation of polar lipids and determination of phospholipids by phosphorus analysis of spots. *Lipids.* 5:494–496.
14. McKeown, A. N., J. L. Naro, …, P. F. Almeida. 2011. A thermodynamic approach to the mechanism of cell-penetrating peptides in model membranes. *Biochemistry.* 50:654–662.
15. Ritz, S., K. Eisele, …, E. K. Sinner. 2010. Cationized albumin-biocoatings for the immobilization of lipid vesicles. *Biointerphases.* 5:FA78–FA87.
16. Schön, P., A. J. García-Sáez, …, P. Schwille. 2008. Equinatoxin II permeabilizing activity depends on the presence of sphingomyelin and lipid phase coexistence. *Biophys. J.* 95:691–698.
17. Leontiadou, H., A. E. Mark, and S. J. Marrink. 2006. Antimicrobial peptides in action. *J. Am. Chem. Soc.* 128:12156–12161.
18. Ladokhin, A. S., W. C. Wimley, and S. H. White. 1995. Leakage of membrane vesicle contents: determination of mechanism using fluorescence requenching. *Biophys. J.* 69:1964–1971.
19. Arbuzova, A., and G. Schwarz. 1999. Pore-forming action of mastoparan peptides on liposomes: a quantitative analysis. *Biochim. Biophys. Acta.* 1420:139–152.
20. Nicol, F., S. Nir, and F. C. Szoka, Jr. 1996. Effect of cholesterol and charge on pore formation in bilayer vesicles by a pH-sensitive peptide. *Biophys. J.* 71:3288–3301.
21. Apellániz, B., S. Nir, and J. L. Nieva. 2009. Distinct mechanisms of lipid bilayer perturbation induced by peptides derived from the membrane-proximal external region of HIV-1 gp41. *Biochemistry.* 48:5320–5331.
22. Apellániz, B., J. L. Nieva, …, A. J. García-Sáez. 2010. All-or-none versus graded: single-vesicle analysis reveals lipid composition effects on membrane permeabilization. *Biophys. J.* 99:3619–3628.
23. Fuertes, G., A. J. García-Sáez, …, J. Salgado. 2010. Pores formed by Baxα5 relax to a smaller size and keep at equilibrium. *Biophys. J.* 99:2917–2925.
24. Matsuzaki, K., O. Murase, …, K. Miyajima. 1996. An antimicrobial peptide, magainin 2, induced rapid flip-flop of phospholipids coupled with pore formation and peptide translocation. *Biochemistry.* 35:11361–11368.
25. Matsuzaki, K., O. Murase, …, K. Miyajima. 1995. Translocation of a channel-forming antimicrobial peptide, magainin 2, across lipid bilayers by forming a pore. *Biochemistry.* 34:6521–6526.
26. Rathinakumar, R., and W. C. Wimley. 2008. Biomolecular engineering by combinatorial design and high-throughput screening: small, soluble peptides that permeabilize membranes. *J. Am. Chem. Soc.* 130:9849–9858.
27. Gregory, S. M., A. Pokorny, and P. F. F. Almeida. 2009. Magainin 2 revisited: a test of the quantitative model for the all-or-none permeabilization of phospholipid vesicles. *Biophys. J.* 96:116–131.
28. Rex, S., and G. Schwarz. 1998. Quantitative studies on the melittin-induced leakage mechanism of lipid vesicles. *Biochemistry.* 37:2336–2345.
29. Nelson, P. 2004. Biological Physics: Energy, Information, Life. Freeman, New York.
30. Tamba, Y., H. Ariyama, …, M. Yamazaki. 2010. Kinetic pathway of antimicrobial peptide magainin 2-induced pore formation in lipid membranes. *J. Phys. Chem. B.* 114:12018–12026.
31. Needham, D., J. Y. Park, …, J. Tong. 2013. Materials characterization of the low temperature sensitive liposome (LTSL): effects of the lipid composition (lysolipid and DSPE-PEG2000) on the thermal transition and release of doxorubicin. *Faraday Discuss.* 161:515–534, 563–589.
32. Huang, H. W. 2000. Action of antimicrobial peptides: two-state model. *Biochemistry.* 39:8347–8352.
33. Sengupta, D., H. Leontiadou, …, S. J. Marrink. 2008. Toroidal pores formed by antimicrobial peptides show significant disorder. *Biochim. Biophys. Acta.* 1778:2308–2317.
34. Lee, M. T., W. C. Hung, …, H. W. Huang. 2008. Mechanism and kinetics of pore formation in membranes by water-soluble amphipathic peptides. *Proc. Natl. Acad. Sci. USA.* 105:5087–5092.
35. Bergstrom, C. L., P. A. Beales, …, J. T. Groves. 2013. Cytochrome *c* causes pore formation in cardiolipin-containing membranes. *Proc. Natl. Acad. Sci. USA.* 110:6269–6274.
36. Matsuzaki, K. 2009. Control of cell selectivity of antimicrobial peptides. *Biochim. Biophys. Acta.* 1788:1687–1692.

Supporting Information

PE and PS Lipids Synergistically Enhance Membrane Poration by a Host-Defense Peptide with Anticancer Properties

Natália Bueno Leite[1], Anders Aufderhorst-Roberts[2], Mario Sergio Palma[3], Simon D. Connell[2], João Ruggiero Neto[1,*], Paul A. Beales[4,*]

Author Affiliations:

[1] Department of Physics IBILCE – São Paulo State University -UNESP;

[2] School of Physics and Astronomy and Astbury Centre for Structural Molecular Biology, University of Leeds, Leeds, UK

[3] Center of Studies of Social Insects/Dept. Biology/ IB – São Paulo State University – UNESP.

[4] School of Chemistry and Astbury Centre for Structural Molecular Biology, University of Leeds, Leeds, UK

Brief derivation of the relationship between membrane permeability and fractional permeable area of membrane.

This follows the arguments previously presented by Philip Nelson (Nelson P. 2004. Biological Physics: Energy, Information, Life (Freeman, New York)).

We consider a cylindrical water channel across of biological membrane of length δ, where δ is the bilayer thickness. Initially the concentration of solute (c) outside of the vesicle is c_0 and inside the vesicle is 0. Solute will diffuse through the pore to equilibrate the concentration gradient, Δc, across the membrane. After a brief initial phase of solute influx, a quasi steady state is reached where $dc/dt = 0$. Given the diffusion equation,

$$\frac{dc}{dt} = D_0 \frac{d^2c}{dx^2}$$

where D_0 is the diffusion constant of the solute. This implies that $d^2c/dx^2 = 0$, where x is the spatial coordinate along the length of the pore, parallel to the bilayer normal. This can be solved with the boundary conditions $c(0) = c_0$ and $c(\delta) = 0$ to give $c = c_0(1 - x/\delta)$. By Fick's law, it then follows that the flux $j = -D_0\,\Delta c/\delta$, which can be written as $j = -P_p\Delta c$, where $P_p = D_0/\delta$ is the permeability of a single pore.

If we consider a larger membrane area that might have multiple pores, the diffusive transport across the membrane will only occur through the fraction of the total membrane area (α) that is porous. Therefore the permeability of the membrane, $P_m = \alpha D_0/\delta$, which can be rearranged to give

$$\frac{A_p}{A_v} = \frac{P_m}{D_0}\delta$$

Where A_p is the total area of pores on a vesicle of area A_v.

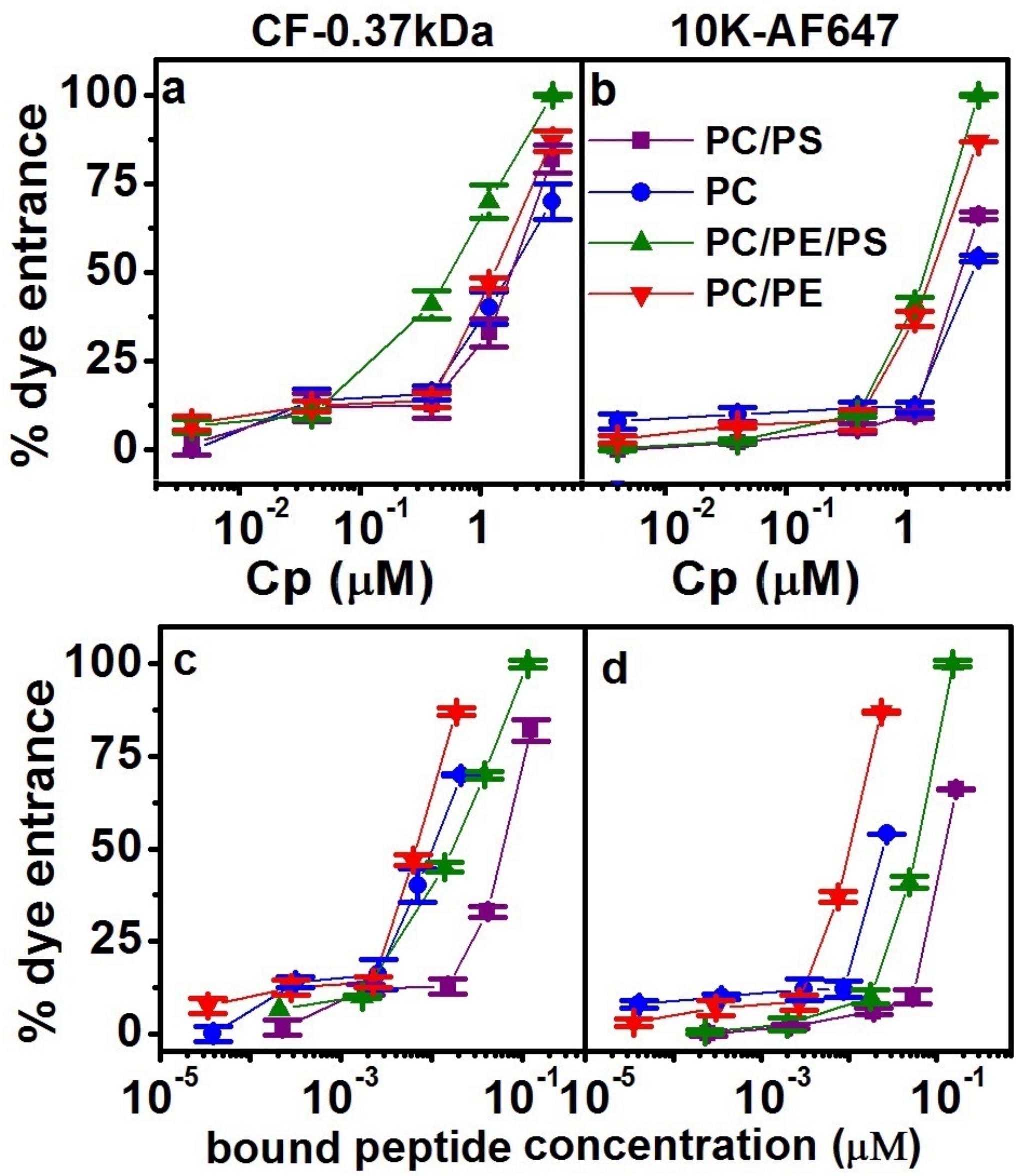

Figure S1. GUV leakage data plotted as average % dye leakage across the GUV population against (a, b) total peptide concentration, and (c, d) peptide concentration bound to the membranes for the different membrane compositions studied. (a, c) leakage of the CF probe; (b, d) leakage of the 10k-AF647 probe. The error bars were obtained through the mean standard deviation of the observed GUVs set.

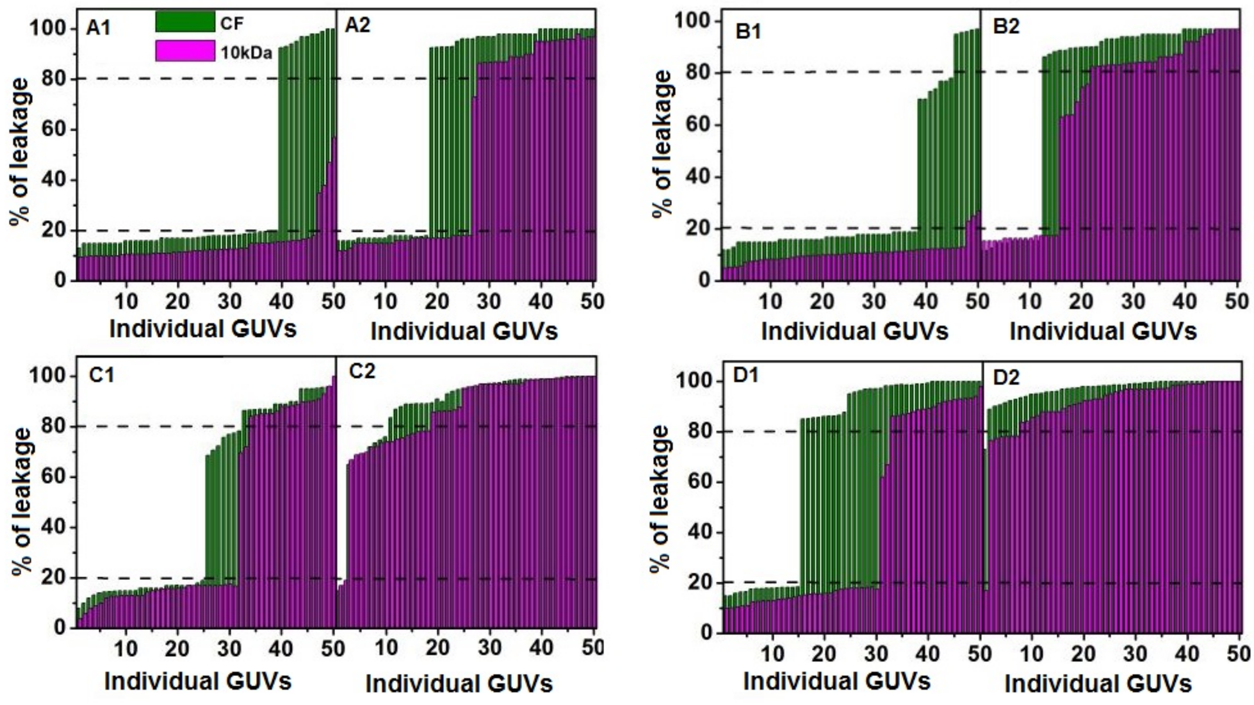

Figure S2 – GUV leakage histograms. Fractional leakage histograms for 50 GUVs per lipid composition after 30 min. incubation with (1) 1.2 µM and (2) 4.0 µM of MP1. Individual GUVs are ordered left to right from lowest to highest leakage %. Lipid compositions are (A) PC, (B) PC/PS, (C) PC/PE and (D) PC/PE/PS. Leakage is analysed for the CF (green) and 10k-AF647 (magenta) probes. Horizontal lines at 20% and 80% leakage represent the thresholds used to categorized leaked vesicles and fully leaked vesicles, respectively. Note that partially leaked vesicles are comparatively rare for these peptide – membrane systems.

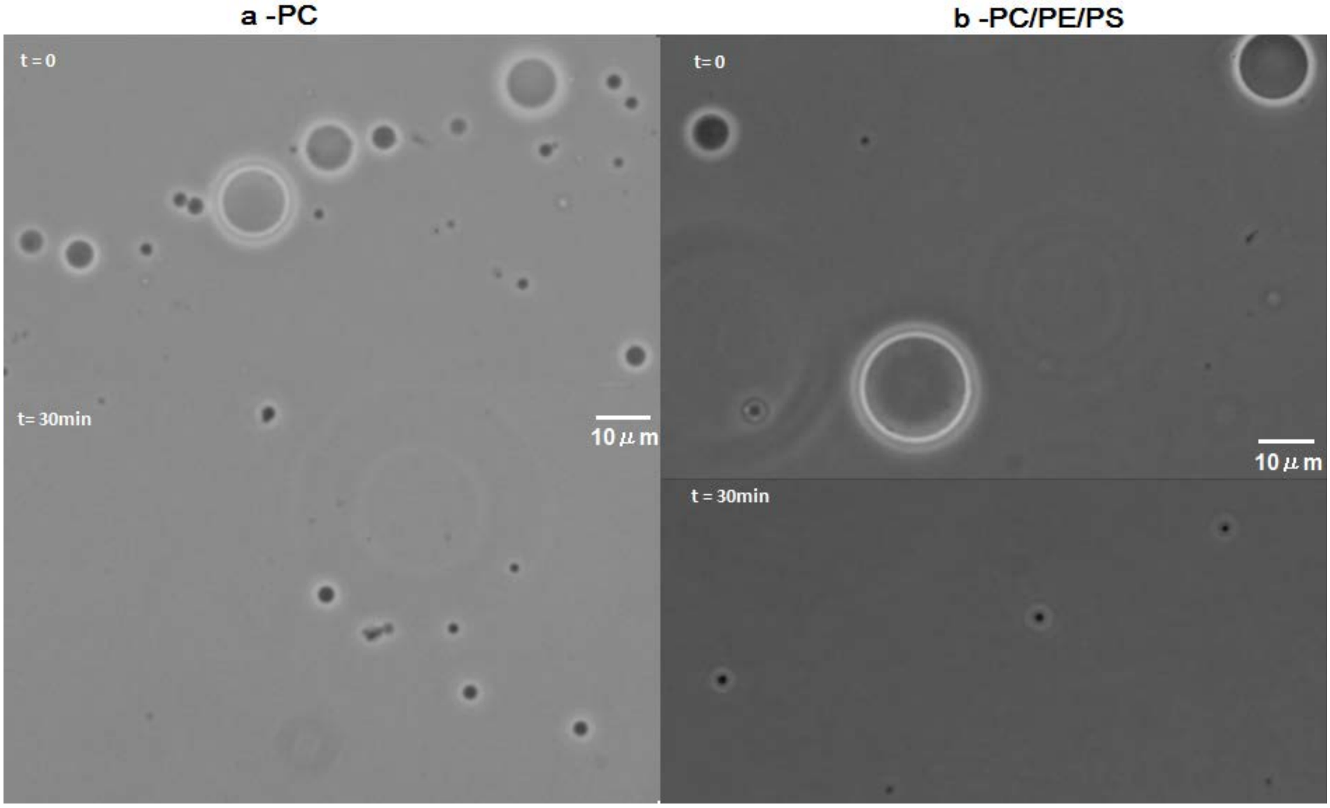

Figure S3 - Phase Contrast of GUVs. Peptide solution was added to GUVs solution to reach a final concentration of 10 µM. Before the peptide addition, at t=0, GUVs were observed in the sample. After the peptide addition, the GUVs are observed to have been completed disrupted (lysed) after 30 min. a- GUVs composed of PC are shown in t=0, and 30 min after peptide addition in the same region of the sample; almost no more GUVs are observed. b- GUVs composed of PC/PE/PS are shown in t=0, and 30 min after peptide addition at the same region; almost no more GUVs are observed.

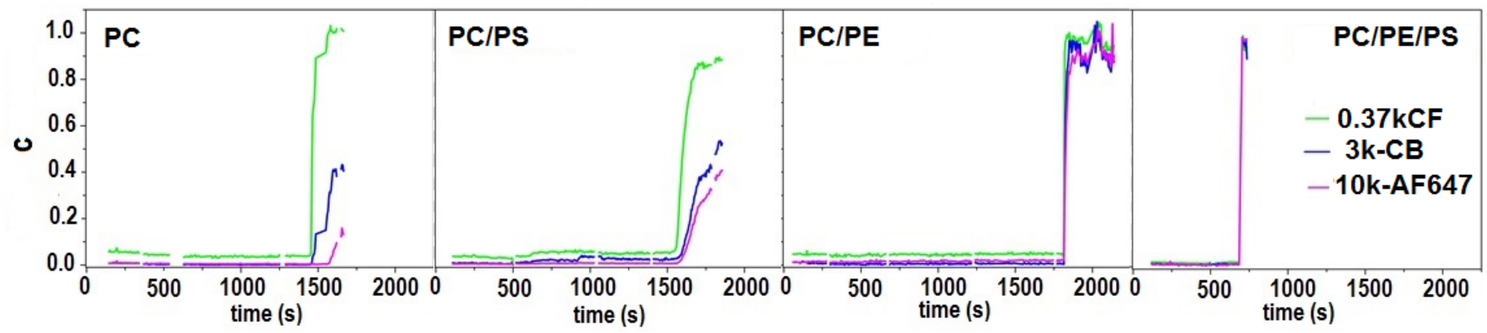

Figure S4. Example profiles for the typical kinetics of single vesicle permeation due to the addition of MP1 at 4.0μM final concentration in four lipid compositions: by PC, PC/PS, PC/PE and PC/PE/PS.

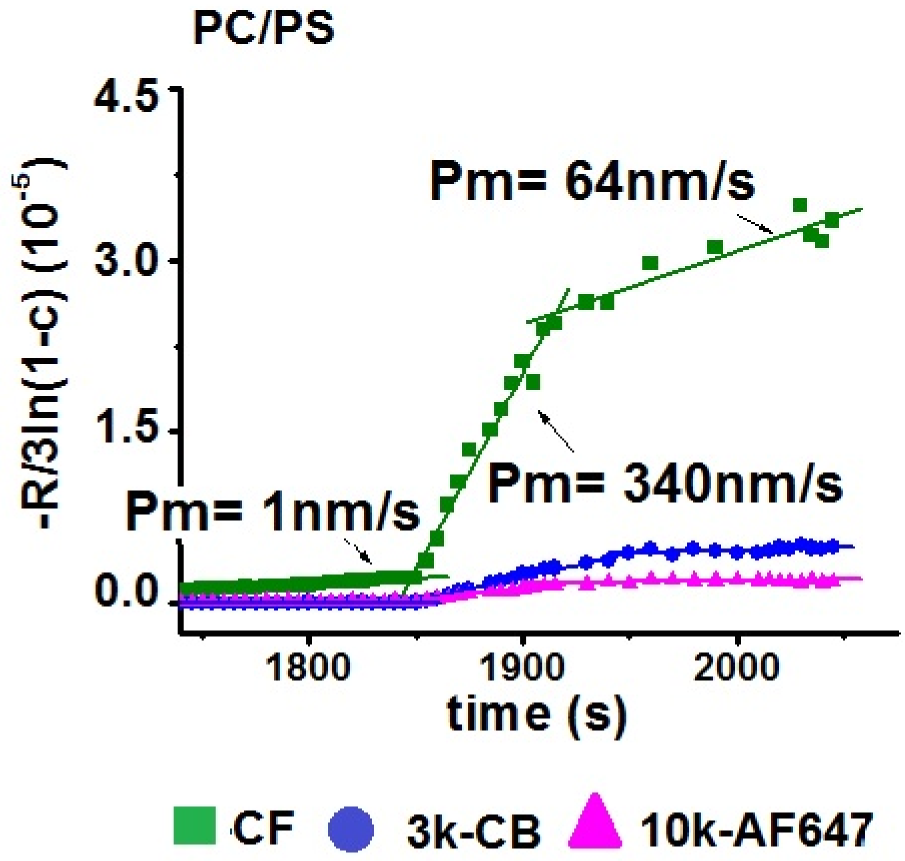

Figure S5. A permeability plot for a PC/PS GUV that clearly exhibits significant changes in membrane permeability during the initial leakage events. The permeability to the CF probe changes dynamically from 1 nm/s to 340 nm/s to 64 nm/s with abrupt transitions between these stages.

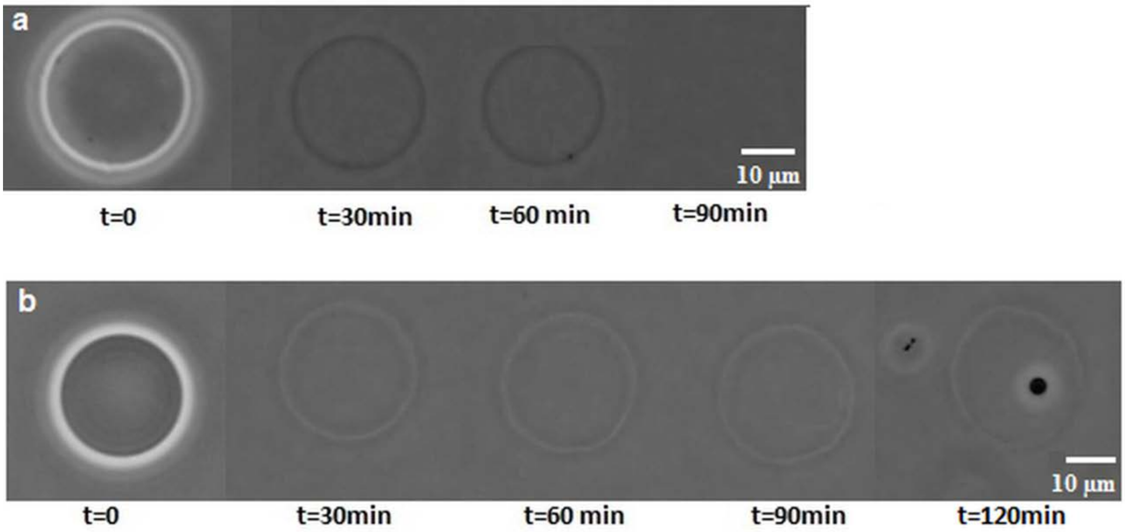

Figure S6 - Phase contrast images of GUVs composed of (a) PC/PE/PS and (b) PC/PS are obtained for a period of 2 h. after addition of 4.0 μM MP1. a- At t=30min most of the phase contrast of the GUV is lost, due to leakage of the encapsulated sucrose solution; the vesicle structure integrity is kept intact for ~1 h. after the contrast is lost. The vesicle size decreased with increasing time of peptide interaction according to: at t ≈ 0 the vesicle diameter (d) was 19 ± 1 µm; at t ≈ 30 min., d= 18 ± 1 µm; at t ≈ 60 min., d = 17 ± 2 µm; and at t ≈ 85 min. the vesicle had lost its integrity. b- At t = 30 min. most of the phase contrast is lost from the GUV and the vesicle structure integrity remains intact over 2 h. after the contrast decreasing. The vesicle size does not changed with increasing time of peptide interaction; the vesicle diameter remained at 25 µm during the observation time.

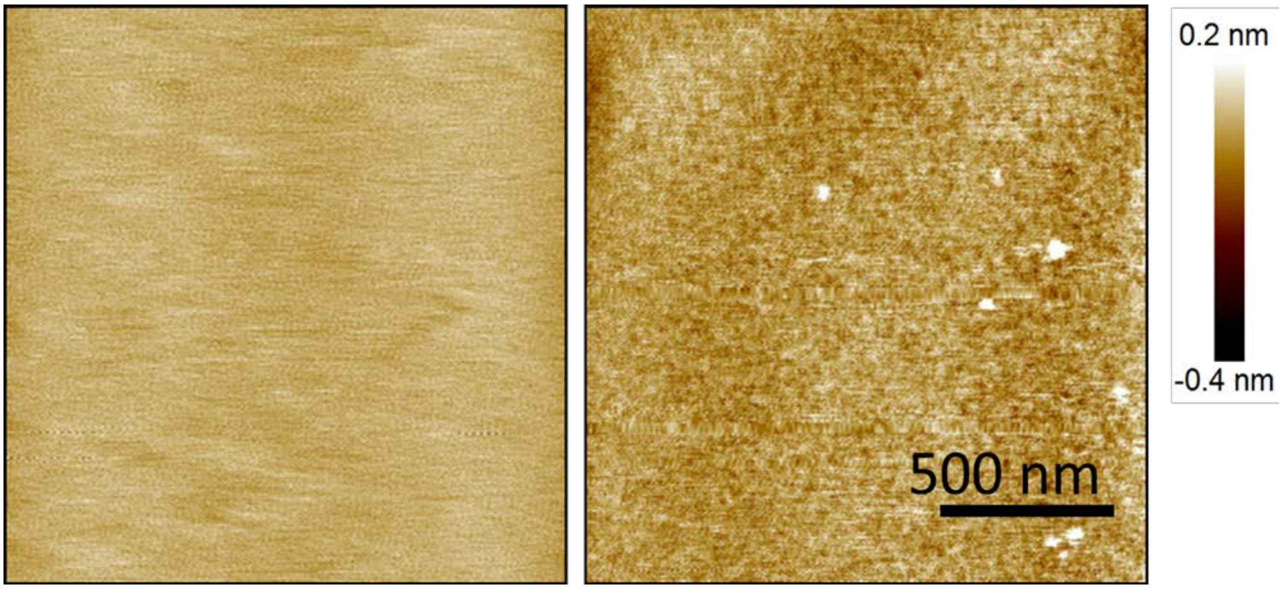

Figure S7. AFM images of a DOPC membrane 80 min. after addition of 10 μM MP1. No clear defects can be observed, but a textured bilayer surface can be seen if the z-contrast is enhanced by a factor of 10. This could indicate a layer of absorbed peptide.

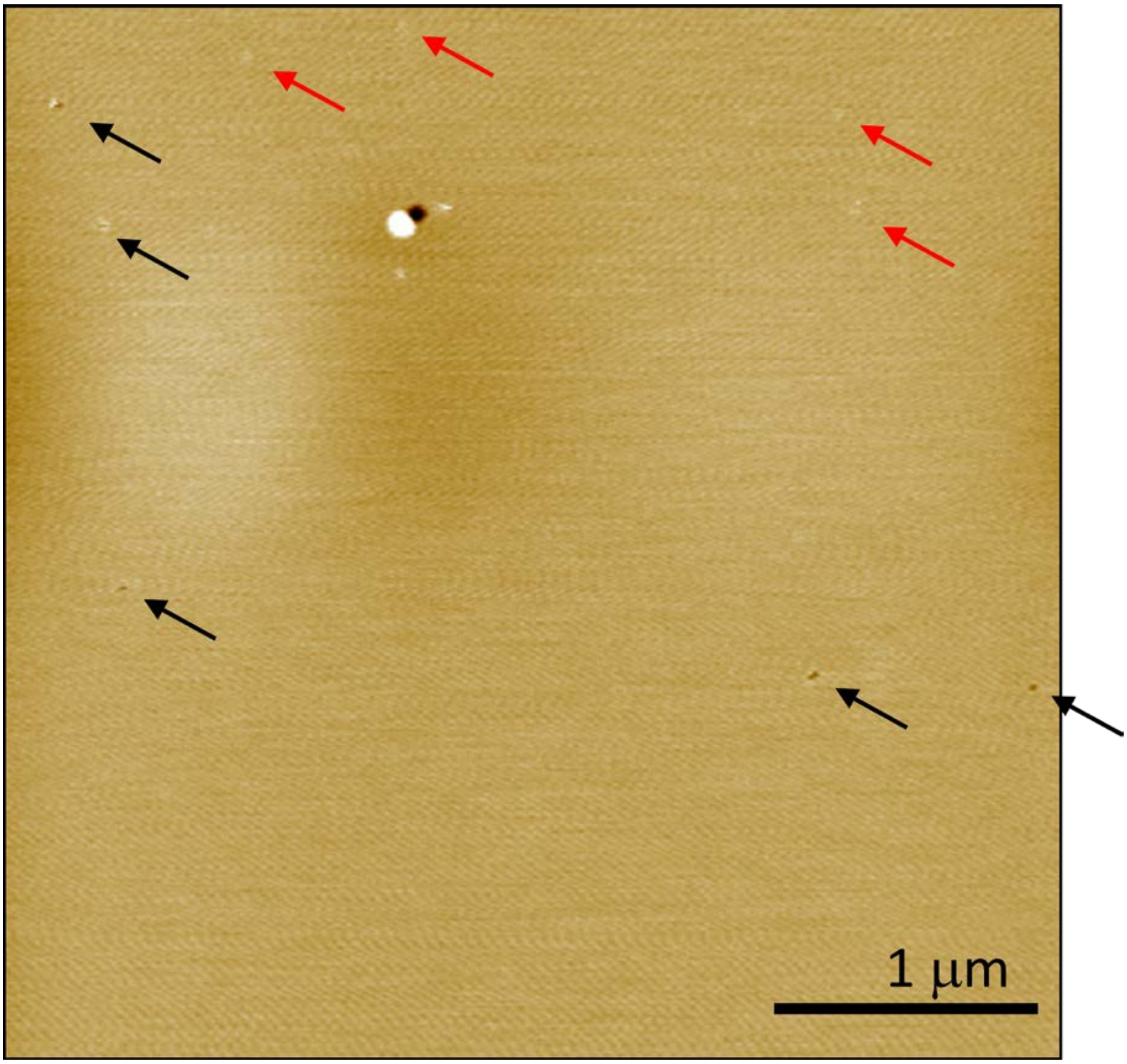

Figure S8. AFM image showing 10-20 nm pin-holes (black arrows) in PC/PE/PS membranes in the first scan, 5 min. after addition of 10 µM MP1. In addition feint "blisters" (some depicted by red arrows) can be seen, which will later develop into pores. A larger pore can be seen top centre-left with a vesicle forming at the edge; vesicle micellisation at the pore rim was observed in time lapse sequences to be the quantized growth mechanism of larger pore defects.

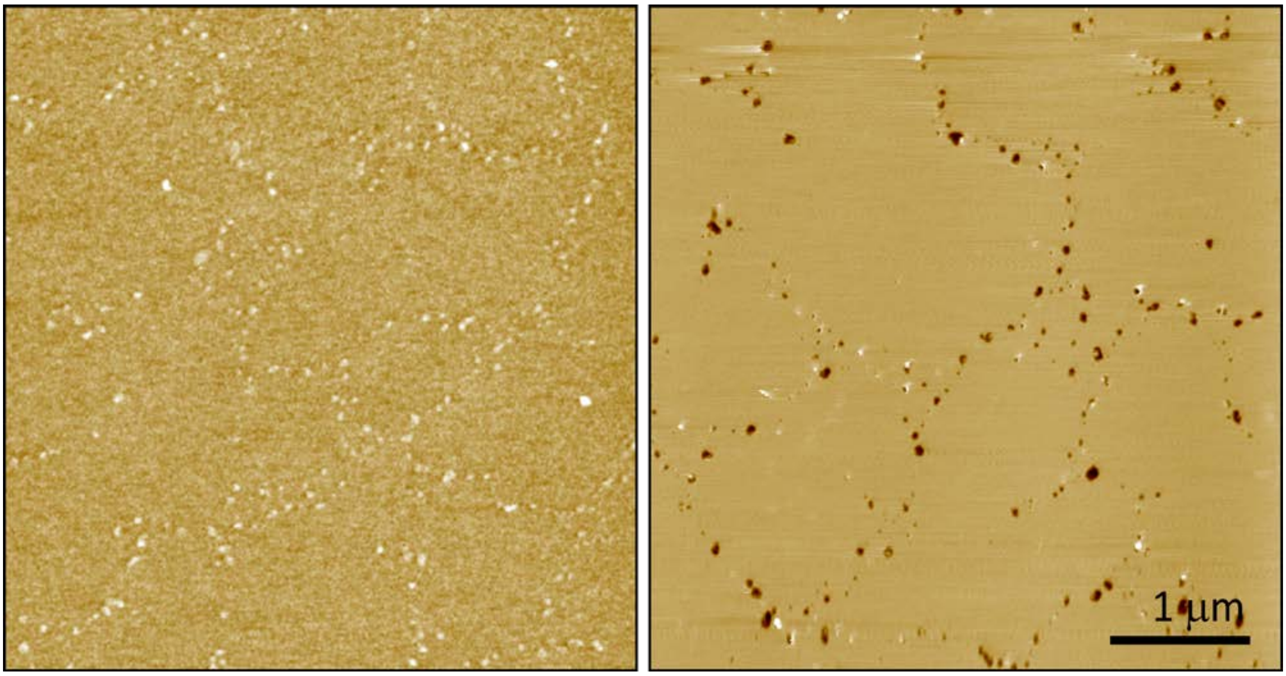

Figure S9. AFM images of PC/PS membranes. (Left) 5-10 min. after addition of 10 μM MP1 "blisters" can be observed in the membrane that will eventually form pores. (Right) Pores observed in the membrane at 2 h after peptide addition. Interestingly, note that the blisters and pores appear to form along a network of lines on the membrane, implying that there may be some cooperativity between the occurrences of neighboring membrane defects.

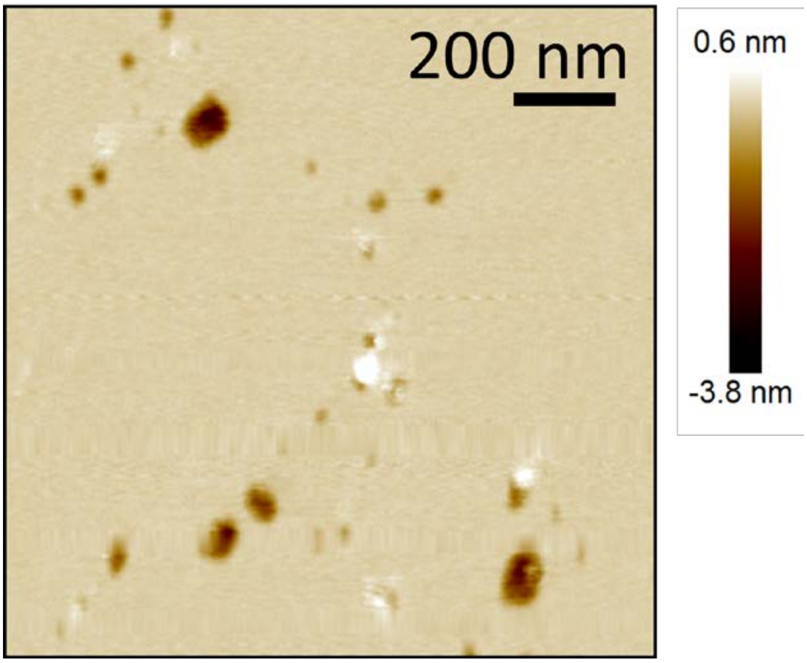

Figure S10. AFM image showing the structural details of pores in PC/PS membranes. Half-bilayer defects (mid-brown) can be observed, along with pores containing full-bilayer pores in their centre (black).
